# HIV-1 Reverse Transcriptase Still Remains a New Drug Target: Structure, Function, Classical Inhibitors, and New Inhibitors with Innovative Mechanisms of Actions

**DOI:** 10.1155/2012/586401

**Published:** 2012-06-20

**Authors:** Francesca Esposito, Angela Corona, Enzo Tramontano

**Affiliations:** Department of Life and Environmental Sciences, University of Cagliari, Cittadella Universitaria di Monserrato, SS 554, 09042 Monserrato, Italy

## Abstract

During the retrotranscription process, characteristic of all retroviruses, the viral ssRNA genome is converted into integration-competent dsDNA. This process is accomplished by the virus-coded reverse transcriptase (RT) protein, which is a primary target in the current treatments for HIV-1 infection. In particular, in the approved therapeutic regimens two classes of drugs target RT, namely, nucleoside RT inhibitors (NRTIs) and nonnucleoside RT inhibitors (NNRTIs). Both classes inhibit the RT-associated polymerase activity: the NRTIs compete with the natural dNTP substrate and act as chain terminators, while the NNRTIs bind to an allosteric pocket and inhibit polymerization noncompetitively. In addition to these two classes, other RT inhibitors (RTIs) that target RT by distinct mechanisms have been identified and are currently under development. These include translocation-defective RTIs, delayed chain terminators RTIs, lethal mutagenesis RTIs, dinucleotide tetraphosphates, nucleotide-competing RTIs, pyrophosphate analogs, RT-associated RNase H function inhibitors, and dual activities inhibitors. This paper describes the HIV-1 RT function and molecular structure, illustrates the currently approved RTIs, and focuses on the mechanisms of action of the newer classes of RTIs.

## 1. Introduction

Since the human immunodeficiency virus (HIV) has been established to be the etiological agent of the acquired immunodeficiency syndrome (AIDS) [1, 2], an originally unpredicted number of drugs have been approved for the treatment of the HIV-infected patients [3]. This success in effective drugs identification, certainly unique in the treatment of viral infections, together with the use of such armamentarium in different combination therapeutic regimens, has transformed a highly lethal syndrome into a chronic disease [4]. The management of this disease, however, is still complex and worrisome due to problems such as monitoring of therapy efficacy, chronic administration drug toxicity, poor tolerability, drug resistance development, or therapy adjustment after treatment failures [4]. For all these reasons, the search for new inhibitors, possibly acting with molecular mechanisms different from the ones of the already approved drugs or anyway showing different patterns of drug resistance and, possibly, with diverse drug-associated chronic toxicity, is still a worldwide health care issue.

The success in HIV infection therapy is certainly related to the fact that the HIV life cycle has been intensely dissected; several of its steps have been validated as drug targets, and, subsequently, a number of viral inhibitors have been identified and developed against many of them [3, 4]. Among the HIV proteins which have been deeply characterized as major drug targets is the reverse transcriptase (RT), the virus coded enzyme that converts the ssRNA viral genome into the dsDNA provirus which is consequently imported into the cell host nucleus and integrated into the host chromosome by another virus-coded protein, integrase (IN). The present paper focuses on the RT function within the virus cycle, its molecular structure, the mechanism of action of the currently approved RT inhibitors (RTIs), and the newer classes of RTIs and their modes of action.

## 2. Retrotranscription Process

**Figure 1:**
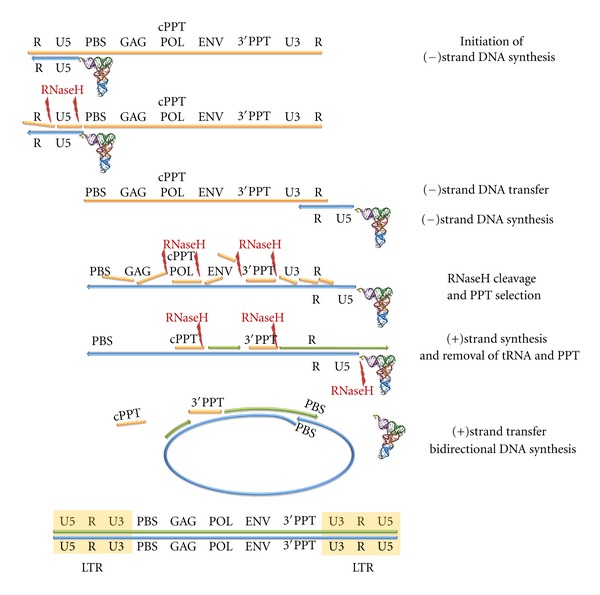
HIV-1 reverse transcription process. Step 1: host cell tRNALys3 hybridizes to the PBS near the 5′-end of the (+)strand RNA genome (orange). (−)strand DNA (blue) synthesis starts using host tRNALys3 as a primer. DNA synthesis proceeds up to the 5′-end of the RNA genome. Step 2: RNase H hydrolysis of the RNA portion of the RNA:DNA hybrid product exposes the ssDNA product determining the (−)strand strong stop DNA. Step 3: strand transfer of the (−)strand DNA through its hybridization with the R region at the 3′-end of the ssRNA genome and further elongation of the (−)strand DNA. Step 4: DNA synthesis proceeds, and the RNase H function cleaves the RNA strand of the RNA:DNA at numerous points leaving intact two specific sequences (cPPT, 3′PPT) resistant to the RNase H cleavage. Step 5: (−)strand DNA synthesis (green) initiation using PPTs as primers. Step 6: RNase H hydrolysis of the PPT segments and the junction of the tRNA:DNA hybrid, freeing the PBS sequence of the (+)strand DNA. Step 8: strand transfer of the PBS sequence of the (+)strand DNA that anneals to the PBS on the (−)strand DNA. DNA synthesis then continues with strand displacement synthesis. Step 9: the product is a linear dsDNA with long terminal repeats (LTRs) at both ends.

After the HIV particle fuses with the host cell surface, the viral particle content is released within the host cell cytoplasm where the viral ssRNA genome serves as template to obtain a proviral dsDNA that is integrated into the host genome, becoming a source of mRNAs coding for viral proteins and ssRNA genomes that, together, will form the new viral particles. The conversion of the viral ssRNA genome into integration-competent dsDNA, termed retrotranscription (Figure 1), is characteristic of all retroviruses, and its accomplishment requires viral as well as cellular elements, among which the most important is the virus-coded RT protein.

Each HIV particle contains two copies of (+)ssRNA genome sequence of 9,7 kb [5] coding for structural and nonstructural proteins and having, in the 5′- and 3′-ends, two identical sequences. Near the 5′-end of the viral genome there is an 18-nucleotides-long segment, termed primer binding site (PBS), which is complementary to the 3′-end 18 nucleotides of the human tRNALys3. When the cellular tRNA is hybridized to the PBS, it serves as an RNA primer, and the RT-associated DNA polymerase function can initiate the first (−)strand DNA synthesis using the viral RNA genome as a template (Figure 1). After tRNA elongation until the ssRNA 5′-end, there is a first (−)strand strong-stop DNA. In fact, the (−)strand DNA synthesis generates an RNA:DNA hybrid that is a substrate for the RT-associated ribonuclease H (RNase H) function which selectively degrades the RNA strand of the RNA:DNA hybrid [6], leaving the nascent (−)strand DNA free to hybridize with the complementary sequence at the 3′-end of one of the two viral genomic ssRNAs. A strand transfer, therefore, occurs from the R region at the 5′-end of the genome to the equivalent R region at the 3′-end (see Figure 1). After this step, termed (−)strand transfer, (−)strand synthesis can continue along the viral RNA starting from its 3′-end. Whilst DNA synthesis proceeds, the RNase H function cleaves the RNA strand of the RNA:DNA at numerous points. Although most of the RNase H cleavages do not appear to be sequence specific, there are two specific purine-rich sequences, known as the polypurine tracts (PPTs), that are resistant to the RNase H cleavage and remain annealed with the nascent (−)strand DNA. These two well-defined sites are located in the central part of the HIV-1 genome. In particular, the 3′-end PPT defines the 5′-end of the viral coding (+)strand DNA synthesis since this PPT serves as primer [7, 8]. The (+)strand DNA synthesis continues to the 5′-end of the (−)strand DNA and uses also the 18-nucleotides PBS sequence of the tRNA as a template. Importantly, the 19th base from the 3′-end of tRNALys3 is a methyl A, and the presence of this modified base blocks the RT, generating a (+)strand strong-stop DNA. Subsequently, the RNase H function cleaves the RNA segment of the tRNA:DNA hybrid, freeing the PBS sequence of the (+)strand DNA and allowing it to anneal to the complementary site near the 3′-end of the extended (−)strand DNA [9]. Then, a bidirectional synthesis occurs to complete a viral dsDNA that has a 90-nucleotides single-stranded flap at the center. This unusual situation is probably solved by host mechanisms, and one candidate for flap removal is the flap endonuclease-1 (FEN-1) [8]. Finally, a specific cleavage removes the PPT primers and exposes the integration sequence to facilitate the insertion of the viral dsDNA into the host chromosome.

## 3. RT Structure and Functions

**Figure 2:**
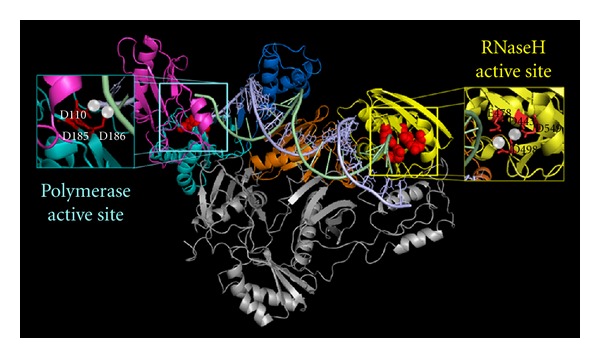
Structure of HIV-1 RT. The enzyme has two domains: the p66 (colored) and the p51 (gray). The polymerase domain shows a characteristic highly conserved structure that resembles a right hand, consisting of fingers domain (magenta), palm domain (cyan), thumb domain (blue). The p66 subunit also comprises the connection domain (orange) and RNase H domain (yellow). The polymerase active site is located in the middle of palm, fingers, and thumb subdomains. The three catalytic aspartic acid residues (D110, D185 and D186) located in the palm subdomain of p66 that bind the cofactor divalent ions (Mg^2+^) are shown (red). The RNase H domain is located at C-terminus of the p66 subunit, 60 Å far from polymerase active site. The RNase H active site contains a DDE motif comprising the carboxylates residues D443, E478, D498, and D549 that can coordinate two divalent Mg^2+^.

As a major target for anti-HIV therapy, RT has been the subject of extensive research through crystal structure determinations, biochemical assays, and single-molecule analyses. RT derives from a virus-coded polyprotein that is processed by the viral protease to give rise to two related subunits of different length, the p66 and the p51, that share a common amino terminus and combine in a stable asymmetric heterodimer [10]. Analysis of the crystal structure of RT reveals that p66 is composed of two spatially distinct domains, polymerase and RNase H domains (Figure 2). The polymerase domain shows a characteristic highly conserved structure that resembles a right hand, consisting of fingers (residues 1–85 and 118–155), palm (residues 86–117 and 156–237), and thumb (residues 238–318) subdomains. The p66 subunit also comprises the connection subdomain (residues 319–426) and RNase H domain (residues 427–560) [11, 12]. The p51 subunit lacks the RNase H domain and has the same four subdomains of the p66 polymerase domain whose relative positions, however, are different. For this reason, the p51 subunit folds differently from p66; it does not have enzymatic activities while it serves to anchor the proper folding of the p66 subunit that performs all the catalytic functions.

RT is primarily responsible for several distinct activities that are all indispensable for the retrotranscription process: RNA- and DNA-dependent DNA synthesis, RNase H activity, strand transfer, and strand displacement synthesis [13]. The presence of all these functions in a single protein is facilitated by the highly dynamic RT nature which allows RT to spontaneously slide over long distances of RNA:DNA and DNA:DNA duplexes, to easily target the primer terminus for DNA polymerization, to rapidly access multiple sites, and, hence, to make up for its low processivity [13]. RT sliding does not require energy from nucleotide hydrolysis, and it is supposed to be a thermally driven diffusion process [13]. Noteworthy, it has been recently shown that RT can bind to the nucleic acid substrates in two different orientations, termed “RNase H cleavage competent orientation” and “polymerase competent orientation,” and that each of them allows to catalyze one of the two RT-associated enzymatic activities [14]. These two binding modes are in a dynamic equilibrium, and it has been demonstrated that RT can spontaneously and rapidly switch between these orientations without dissociating from the substrate. This flipping can be influenced by the presence of small molecules as nucleotides that stabilize the polymerase competent orientation or inhibitors that, conversely, destabilize it [8]. Together, shuttling and switching give rise to a very complex series of conformational changes that increase enormously the replication efficiency, combining DNA polymerization and RNA cleavage. 

### 3.1. RNA- and DNA-Dependent DNA Synthesis

The DNA synthesis, catalyzed by both RT-associated RNA- and DNA-dependent DNA polymerase activities (RDDP and DDDP, resp.), occurs with a mechanism that is similar to other DNA polymerases [15]. The polymerase active site is located in the middle of the palm, fingers, and thumb subdomains. In particular, the palm subdomain is very important for positioning of the primer terminus in the correct orientation for nucleophilic attack on an incoming dNTP [16]. Three aspartic acids residues (D110, D185, and D186) located in the palm subdomain of p66 bind the divalent ion cofactor (Mg^2+^) through their catalytic carboxylates group, and are essential for catalysis (Figure 2) [17]. DNA synthesis requires that RT binds to the template:primer on the priming binding site; this interaction is stabilized by a change of the conformation of the p66 thumb (from close to open). Then, the dNTP binds at the nucleotide binding site to form an RT:DNA:dNTP ternary complex [18]. Afterwards, a conformational change of the fingers traps the dNTP, precisely aligning the *α*-phosphate of the dNTP and the 3′-OH of the primer inside of polymerase active site (this is actually the rate limiting step). Under these conditions, the enzyme catalyzes the formation of a phosphodiester bond between the primer 3′-OH and the dNMP with the release of a pyrophosphate. Then, the pyrophosphate is free to go out of the catalytic site. Finally, translocation of the elongated DNA primer frees the nucleotide-binding site for the next incoming dNTP or, alternatively, RT can dissociate from the complex. Compared to cellular DNA polymerases, RT exhibits a very low processivity, typically dissociating from the substrate after synthesizing only a few to a few hundred nucleotides. This may contribute to the fidelity of RT and results in the accumulation of mutations during reverse transcription. 

Importantly, during its DNA polymerase activity RT can run up against several template secondary structures. Particularly, the RNA template can form stable RNA:RNA interactions that can occlude the polymerization site and/or displace the primer terminus. In this case, RT has been shown to realize a strand displacement synthesis, in which the sliding movement can contribute to the reannealing of the primer, displacing the RNA [17].

### 3.2. DNA-Directed RNA Cleavage

RT is able to degrade selectively the RNA portion of an RNA:DNA hybrid and to remove the priming tRNA and PPT. This RNase H function is essential for virus replication since RNase H-deficient viruses are noninfectious [19]. The RNase H domain is located at C-terminus of the p66 subunit, 60 Å far from polymerase active site (Figure 2) equivalent to 17 nucleotides of a DNA:DNA hybrid and/or 18 nucleotides of a RNA:DNA hybrid [20]. The RNase H active site contains a highly conserved, essential, DDE motif comprising the carboxylates residues D443, E478, D498, and D549, that can coordinate two divalent Mg^2+^ cations, consistently with the proposed phosphoryl transfer geometry [21]. Mutations in any of the D443, D498, and E478 residues abolish enzyme activity [22, 23]. The RNase H domain can catalyze a phosphoryl transfer through nucleophilic substitution reactions on phosphate ester. This action occurs through the deprotonation of a water molecule, with the production of a nucleophilic hydroxide group that attacks the scissile phosphate group on the RNA previously activated by coordination with the Mg^2+^ cofactor [24]. The reason for the RNase H cleavage specificity for the RNA portion of the RNA:DNA hybrid mainly relies on its particular minor groove width and its interaction with the “primer grip” (an extensive network of contacts between the hybrid phosphate backbone and several residues far *∼*4–9 bp from the RNase H active site) [16]. The RNA:DNA hybrid has a minor groove width of *∼*9-10 Å, that is intermediate between the A- and B-form of other double-stranded nucleic acids (dsNA). The HIV-1 RNase H hydrolyzes much less efficiently hybrids with lower widths, such as the PPTs that show a width of 7 Å probably due to the presence of A-tracts [17, 25]. This fact allows the PPT recognition as RNA primers for DNA synthesis and may also represent a further specific viral target.

The RNase H catalysis can occur in a polymerase-dependent or polymerase-independent mode, and it is possible to distinguish three different cleavage types: “DNA 3′-end-directed cleavage,” “RNA 5′-end-directed cleavage,” and “internal cleavage” [26]. The former acts during (−)strand DNA synthesis, when the RNase H active site cleaves the RNA in a position based on the binding of the polymerase active site to the 3′-end of the new (−)DNA [27]. The second one acts when RT binds to a recessed RNA 5′-end annealed to a longer DNA strand, and the RNase H function cleaves the RNA strand 13–19 nucleotides away from its 5′-end. The internal cleavage occurs since the RNA cleavage is slower than DNA synthesis, and, given that a viral particle contains 50–100 RTs molecules and only two copies of (+)RNA, all the nonpolymerizing RTs can bind to the hybrid and degrade the RNA segment by a polymerase-independent mode [16].

### 3.3. Strand Transfer

The strand transfer is a critical step during the reverse transcription process in which two complementary ssNAs have to anneal to allow the pursuance of DNA synthesis (Figure 1). In both (−) and (+)strand transfers the ssNA develops secondary structures: the R region consists of a strong-structured motif TAR hairpin and a poly(A) hairpin [28]. Also the PBS sequence at the 3′-end of the (−)strand DNA can form a stable hairpin structure. Therefore, RT is helped in performing this step by the presence of the viral-coded nucleocapsid (NC) protein [29, 30]. The strand transfer process, together with the RT fidelity and the presence of other host factors such as APOBEC [31], helps to explain the high rate of recombination events to allow HIV to evolve rapidly and develop resistance to drugs.

### 3.4. Pyrophosphorolysis

As most DNA polymerases, RT can catalyze the reversal of the dNTP incorporation that is termed pyrophosphorolysis. RT has the ability to carry out this reverse reaction using a pyrophosphate (PPi) molecule or an NTP, such as ATP, as the acceptor substrate [32–34] giving rise to a dinucleotide tetraphosphate (formed by the excised dNMP and the acceptor ATP substrate) and a free 3′-OH end as reaction products. This RT function is particularly important, as discussed later, in some drug resistance mechanisms.

## 4. Current RTIs: Structure, Mode of Action, and Resistance

The approved combination treatments used for HIV-1 include two classes of RTIs that target the viral enzyme with two different mechanism of action. The first class comprises compounds known as nucleoside/nucleotide RT inhibitors (NRTIs/NtRTIs), while the second class comprises compounds known as nonnucleoside RT inhibitors (NNRTIs).

### 4.1. Nucleoside RT Inhibitors

**Figure 3:**
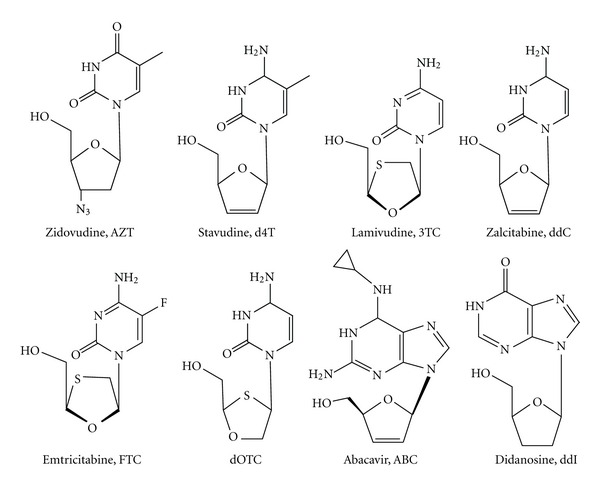
Chemical structures of approved NRTIs.

**Figure 4:**
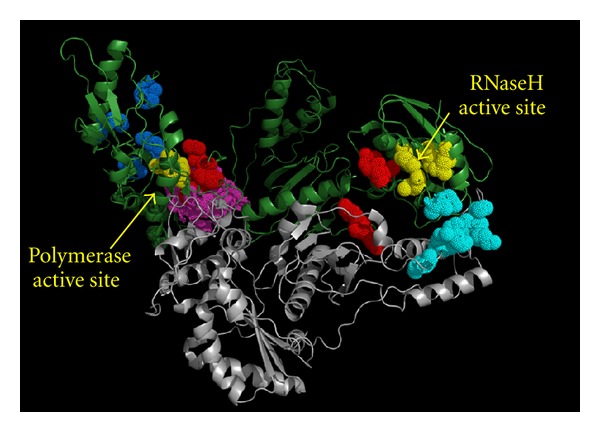
Amino acid residues involved in RTI binding. RT two subunits are in green (p66) and in gray (p51). The catalytic residues of the polymerase active site and the RNase H active site are colored in yellow. NRTIs and NtRTIs interact with residues close to the polymerase active site (blue). NNRTIs bind in a hydrophobic pocket next to the polymerase active site (magenta). RHRTIs such as DKAs, N-hydroxyimides, N-hydroxy quinazolinediones and naphthyridine derivatives bind in the RNase H active site (in yellow on the right). Vinylogous ureas bind to a hydrophobic pocket at the interface between the RNase H domain and the p51 subunit (cyan). Hydrazone derivatives have been proposed to bind two different sites (red). One located between the polymerase active site and the NNRTI-binding pocket (sharing a few residues with it) and the second one located between the RNase H and the connection domain. Anthraquinone derivatives have been proposed to bind to the first hydrazone pocket next to the NNRTI-binding site.

**Figure 5:**
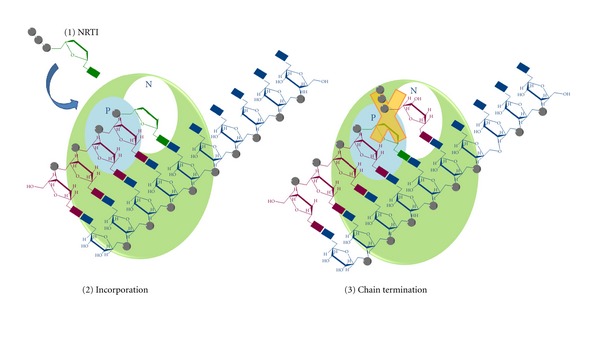
Mechanism of action of RT inhibitors acting as chain terminators. The RT is represented as a pale green circle with the priming binding site in cyan (P) and the nucleotide binding site in white (N).  The RNA template is showed in blue and the (−)strand DNA in purple. The NRTI triphosphate (strong green) (1) competes for the binding with the natural dNTPs, it is incorporated into the growing DNA (2) and it blocks the further DNA elongation because it lacks the 3′-hydroxyl group (3).

There are currently eight NRTIs clinically available, structurally resembling both pyrimidine and purine analogues [3]. Pyrimidine nucleoside analogues include thymidine analogues such as 3′-azido-2′,3′-dideoxythymidine (zidovudine, AZT), and 2′,3′-didehydro-2′,3′-dideoxythymidine (stavudine, d4T) and cytosine analogues such as (−)-2′,3′-dideoxy-3′-thiacytidine (lamivudine, 3TC), 2′,3′-dideoxycytidine (zalcitabine, ddC) which, however, is no longer recommended due to peripheral neuropathy [35], (−)-2′,3′-dideoxy-5-fluoro-3′-thiacytidine (emtricitabine, FTC), and [(−)-2′-deoxy-3′-oxa-4′-thiacytidine) (dOTC). Purine nucleoside analogues include (IS-4R)-4-[2-amino-6(cyclopropylamino)-9H-purin-9yl]-2-cyclopentane-I-methanol (abacavir, ABC) and 2′,3′-dideoxyinosine (didanosine, ddI) as guanosine and adenine analogues, respectively (Figure 3) [3]. These agents, in order to inhibit reverse transcription, have to be phosphorylated by cellular kinases to their triphosphate derivatives. All NRTIs follow the same mechanism of RT inhibition: once activated to their triphosphate form, they are incorporated by RT into the growing primer (Figure 4), competing with the natural dNTPs and terminating DNA synthesis due to their lack of the 3′-hydroxyl group (Figure 5). Therefore, once incorporated into dsDNA they prevent the incorporation of the incoming nucleotide. Importantly, while HIV-1 RT uses these NRTIs as substrates, the cellular DNA polymerases do not recognize them with the same affinity.

Under selective drug pressure, drug resistant viral mutants can gain a competitive advantage over wt virus and become the dominant quasispecies. HIV-1 resistance to NRTIs usually involves two general mechanisms: NRTI discrimination, that reduces the NRTI incorporation rate, and NRTI excision that unblocks NRTI-terminated primers. A simple example of discrimination is steric hindrance in which there is a selective alteration of the NRTI binding and/or incorporation rate such as in the case of the M184V mutation and 3TC [36, 37], where the valine substitution makes steric contacts with the sulfur of the oxathiolane ring of 3TC triphosphate, preventing its proper positioning for catalysis [38]. Even though the discrimination mechanism is less obvious for other NRTIs, in which structurally poorer compounds (e.g., the ones just lacking the 3′-OH group) should be differentially recognized, mutations in the nucleoside-binding site such as K65R, T69D, L74V, V75T, located in the *β*3-*β*4 loop of the p66 fingers subdomain, have been reported to allow a better RT discrimination between NRTI triphosphates and natural dNTPs, since they are involved in the RT interaction with the incoming dNTP [39, 40]. Differently, M41L, D67N, D70R, L210W, T215F/Y, and K219Q mutations, located around the dNTP-binding pocket and also termed thymidine analogs mutations (TAMs), increase NRTI excision. In particular, D67N and K70R are the most important in the excision of 3′-end NRTI-terminated DNA while T215F/Y may increase the RT affinity for the excision substrate ATP so that the NRTI excision is reasonably efficient at ATP physiological concentrations [32, 40, 41]. Other TAMs such as M41L and L210W may stabilize the 215F/Y interaction with the dNTP-binding pocket [42], whereas the K219Q mutation may increase the RT processivity to compensate the higher rate of 3′-nucleotide removal [32, 34]. Recently, mutations in the connection and RNase H domains have also been shown to confer NRTI resistance [43–47]. In particular, connection mutations such as E312Q, G335C/D, N348I, A360I/V, V365I, and A376S have been shown to increase AZT resistance up to 500-fold in the context of TAMs by reducing RNase H activity [43]. This RNase H-dependent mechanism of NRTI resistance has been proposed to be due to an increase in NRTI excision determined by a reduction of RNase H activity [44]. In contrast, the connection mutation G333D, in the context of TAMs and M184V mutation, increases discrimination against 3TC-MP incorporation [48], suggesting an RNase H-independent mechanism of NRTI resistance probably due to long-range interactions and conformational changes in the connection domain [49].

### 4.2. Nucleotide RT Inhibitors

**Figure 6:**
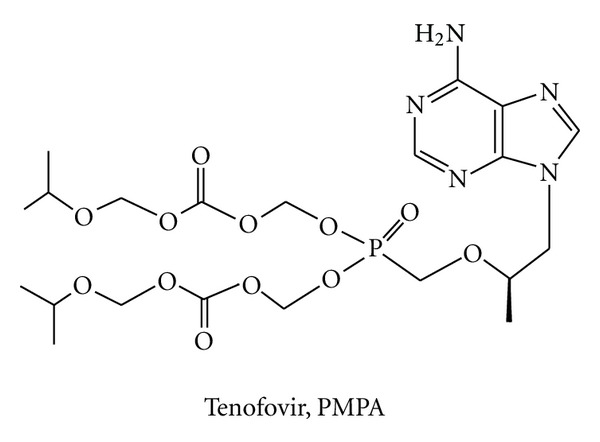
Chemical structure of approved NtRTI.

NtRTIs, such as (R)-9-(2phosphonylmethoxypropyl)-adenine (tenofovir, PMPA) (Figure 6), are compounds that already have a phosphonate group resistant to hydrolysis [3]. Therefore, they only need two phosphorylation steps to be converted to their active diphosphate derivatives, abbreviating the intracellular activation pathway and allowing a more rapid and complete conversion to the active agent [50, 51]. Similarly to NRTIs, NtRTIs are phosphorylated to the corresponding diphosphates by cellular enzymes and serve as alternative substrates (competitive inhibitors); once incorporated into the growing viral DNA, they act as obligatory chain terminators [50]. NtRTIs such as tenofovir are taken as prodrugs to facilitate penetration of target cell membranes. Subsequently, endogenous chemolytic enzymes release the original nucleoside monophosphate analogue that exerts its action [51].

### 4.3. Nonnucleoside RT Inhibitors

**Figure 7:**
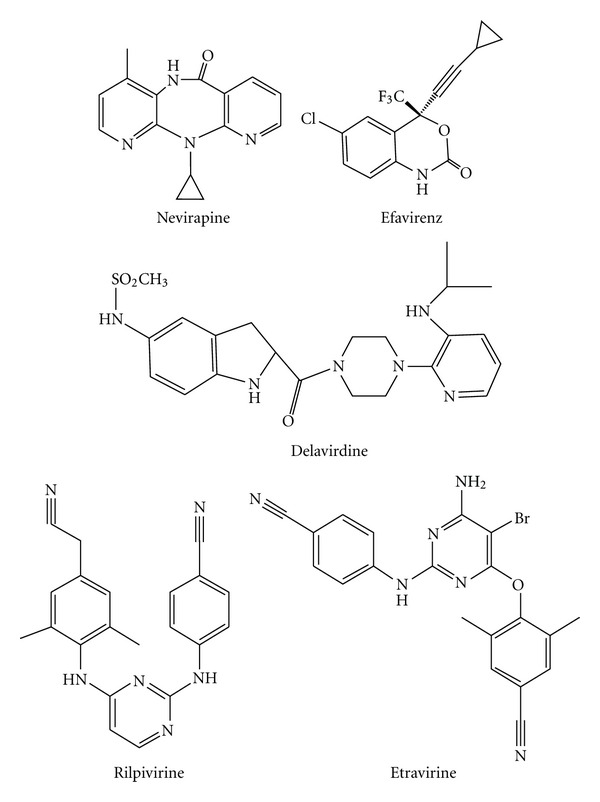
Chemical structures of approved NNRTIs.

NNRTIs are structurally and chemically dissimilar compounds that bind in noncompetitive manner to a hydrophobic RT pocket close to the polymerase active site (Figure 4), distorting the protein and inhibiting the chemical step of polymerization [3, 52]. In fact, NNRTIs binding to RT induces rotamer conformational changes in some residues (Y181 and Y188) and makes the thumb region more rigid, blocking DNA synthesis. Importantly, unlike NRTIs, NNRTIs do not require intracellular metabolism to exert their activity. More than thirty different classes of compounds could be considered to be NNRTIs [3]. The currently approved NNRTIs are 11-cyclopropyl-4-methyl-5H-dipyrido[3,2-b:2′,3′-e][1,4]diazepin-6(11H)-one (nevirapine), (S)-6-chloro-4-(cyclopropylethynyl)-4-(trifluoromethyl)-1H-benzo[d][1,3]oxazin-2(4H)-one (efavirenz), N-(2-(4-(3-(isopropylamino)pyridin-2-yl)piperazine-1-carbonyl)indolin-5-yl)methanesulfonamide (delavirdine) and 4-((6-amino-5-bromo-2-((4-cyanophenyl)amino)pyrimidine-4-yl)oxy)-3,5-dimethylbenzonitrile (etravirine) and 4-((4-((4-(cyanomethyl)-2,6-dimethylphenyl)amino)pyrimidin-2-yl)amino)benzonitrile (rilpivirine) (Figure 7).

Crystallography, molecular modeling and docking studies have revealed that first generation NNRTIs assume a butterfly-like conformation [53–57]. The stabilization of the NNRTI binding in the allosteric site is accomplished through (i) stacking interactions between the NNRTIs aromatic rings and the side chains of Y181, Y188, W229, and Y318 residues in the RT lipophilic pocket; (ii) electrostatic forces (particularly significant for K101, K103, and E138 residues); (iii) van der Waals interactions with L100, V106, V179, Y181, G190, W229, L234, and Y318 residues; (iv) hydrogen bonds between NNRTI and the main chain (carbonyl/amino) peptide bonds of RT [53, 54, 58, 59]. Larger first-generation inhibitors, such as delavirdine, extend towards the flexible loop containing the P236 residue, while maintaining stacking interactions with the tyrosine residues 181 and 188 and hydrogen bonding with K103 [60]. Stacking interactions are less important in the case of efavirenz binding, while hydrogen bonds between the inhibitor and the protein backbone of K101 and K103 residues are critical [61].

First-generation NNRTIs, such as nevirapine and delavirdine, easily select resistant RTs that contain single amino acid mutations such as Y181C, K103N, and Y188C [62, 63], that change their key hydrophobic interactions at the NNRTI binding site. Second-generation NNRTIs, such as efavirenz and dapivirine, usually require two or more mutations in the HIV-1 RT before significantly decreasing their antiviral potency. In general, two or more HIV-1 RT mutations are clustered in the NNRTI pocket, suggesting a direct stereochemical mode of reduction of NNRTI binding, even though other mechanisms may also be present such as the one shown by V108I mutation that induces resistance by perturbing the Y181 and Y188 residues [61] or the one proposed for K103N mutation that should stabilize the apo-RT conformation and, hence, create an energy barrier to NNRTIs binding, reducing their potency [61]. Interestingly, NRTI-resistant mutant virus strains keep full sensitivity to the inhibitory effects of NNRTIs, and vice versa. Recently, however, mutations in the connection and RNase H domains such as N384I, T369I, and E399D have been shown to confer resistance to both NRTIs and NNRTIs probably by altering the template:primer positioning [44, 47, 64].

## 5. New Nucleoside RT Inhibitors

The NRTIs therapeutic use is limited by several factors [65]. Firstly, drug-drug interactions with other NRTIs used in combination treatments such as the one observed between AZT and D4T, that share the same phosphorylation pathway and show a less than additive effect when used in combination [66], or between ddI and tenofovir which determine an increase in single drugs toxicity [65]. Secondly, drug-drug interactions with other molecules such as the one observed when ABC or tenofovir is administered with some protease inhibitors [65, 67], or when ABC is administered with ethanol [68]. Thirdly, several adverse events such as mitochondrial toxicity (linked to myopathy, cardiomyopathy, anemia, lipoatrophy), drug hypersensitivity reactions, and renal dysfunctions have been associated with NRTI treatment [65]. Fourthly, as described above, the selection of NRTI-resistant strains, which is still the main limitation in view of the need for life-long antiviral treatments. Particularly, it has been reported that almost 50% of the viremic patients actually harbor M184V RT mutant strains and that 6–16% of the patients have been infected with viruses resistant to at least one drug and, hence, have a poorer response to therapy and a lower barrier to select further drug-resistant strains [65, 69]. Given this scenario, the new NRTIs which are currently under investigation are sought to have a favorable resistance profile, reduced adverse effects, and/or a novel mechanism of action.

### 5.1. Nucleoside RT Inhibitors in Development Acting as Chain Terminators

**Figure 8:**
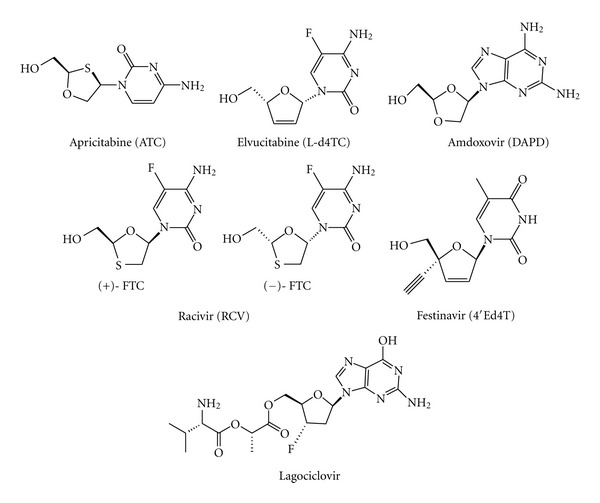
Chemical structures of new NRTIs acting as chain terminators.

(−)-2′-deoxy-3′-oxa-4′-thiocytidine (Apricitabine, ATC) (Figure 8) is a (−)enantiomer deoxycytidine analog with a favorable resistance profile. In fact, ATC shows only a 2-fold potency reduction on TAM strains, with or without the M184V mutation, and on K65R mutant strain, while it shows a 10-fold potency reduction on Q151M mutant strains [70–72]. ATC has a favorable toxic profile with little effects on mitochondrial DNA levels [73], while it shows negative drug-drug interactions when administered with 3TC or FTC [74]. Overall, ATC seems to be a good candidate in NRTI-experienced patients including individuals who have experienced virological failure on 3TC and FTC containing regimens or harboring M184V mutant strains. In fact, ATC has successfully completed the primary endpoint of a phase IIb trial in drug-resistant HIV patients with the M184V mutation.

L-*β*-2′,3′-didehydro-2′,3′-dideoxy-5-fluorocytidine (Elvucitabine, L-d4TC) (Figure 8) is an L-cytidine analog under investigation in phase I/II clinical trials that is more potent than 3TC and that shows no mitochondrial toxicity [75] and an interesting protecting effect on the mitochondrial toxicity due to other NRTIs [76]. L-d4TC resistance profile shows that it selects for M14V RT mutants [77] and has a 10-fold potency reduction on K65R mutant strains [78].

1-*β*-D-2,6-diaminopurine dioxolane (Amdoxovir, DAPD) (Figure 8) is a prodrug under investigation in phase II clinical trials which is deaminated to 1-*β*-D-dioxolane guanosine (DXG) that, upon triphosphorylation, is the active drug. DAPD has a favorable resistance profile since it shows minimal resistance to TAM- and M184V-resistant strains [79, 80], while it shows a >10-fold potency reduction on K65R and Q151M strains [81]. While DAPD, *in vitro*, reduces the mitochondrial DNA content, DXG does not affect it [82].

(±)-*β*-2′,3′-dideoxy-3′-thia-5-fluorocytosine (Racivir, RCV) (Figure 8) is a racemic mixture of (+) and (−)FTC currently under evaluation in phase II/III clinical trials as part of a combination therapy. While both molecules inhibit RT [83], (−)FTC is better phosphorylated than (+)FTC in cells [84], and, therefore, it shows a higher potency in virus inhibition [85]. The RCV resistance profile is interesting; in fact, (−)FTC selects for M184V-resistant strains, while (+)FTC selects for T215Y-resistant strains [86]. Since the simultaneous selection of these two amino acid mutations is incompatible, such racemic mixture orthogonal resistance profile determines a delay in the onset of the drug resistance selection [87]. The long-term mitochondrial toxicity, however, is still to be fully assessed since (+)FTC triphosphate is only 36-fold selective for RT versus DNA polymerase *γ* [88].

In addition, the chain terminator NRTIs Festinavir (4′-Ed4T) [89] and Lagociclovir [90] (Figure 8) are currently under development.

### 5.2. Nucleoside RT Inhibitors with Innovative Mode of Action

The RT inhibition by NRTIs can also be achieved by mechanisms different from the classical chain termination due to the lack of a 3′-hydroxyl group. In particular, new classes of inhibitors with new modes of action are the translocation-defective RT inhibitors (TDRTI), the delayed chain terminators RT inhibitors (DCTRTI), the lethal mutagenesis RT inhibitors (LMRTI), and the dinucleotide tetraphosphates (N_p4_Ns).

#### 5.2.1. Translocation-Defective RT Inhibitors

**Figure 9:**
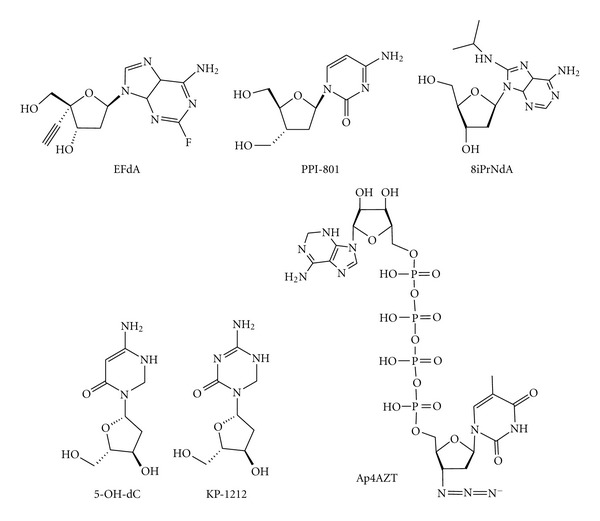
Chemical structures of NRTIs with new mechanisms of action.

**Figure 10:**
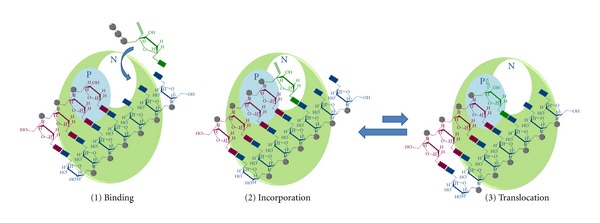
Mechanism of action of TDRTIs. The RT is represented as a pale green circle with the priming binding site in cyan (P) and the nucleotide binding site in white (N). The RNA template is shown in blue and the (−)strand DNA in purple. The TDRTI triphosphate (strong green) can be used as RT substrate (1) and is incorporated in the nucleic acid (2). The incorporated TDRTI blocks the further DNA synthesis since the enzyme is not able to efficiently translocate (3).

TDRTIs are NRTIs with modifications of the sugar moiety that block the RT translocation after the NRTI incorporation. 4′-ethynyl-2-fluoro-2′-deoxyadenosine (EFdA) (Figure 9) is the most potent derivative of a series of 4′-substituted nucleoside analogs which, differently from the other NRTIs, have a 3′-hydroxyl group [91]. EFdA is able to inhibit many drug-resistant strains several orders of magnitude more potently than the other approved NRTIs. For instance, it inhibits the M184V mutant strain with an EC_50_ value of 8 nM, while some other drug-resistant strains are even hypersensitive to EFdA [92]. Importantly, RT can use EFdA triphosphate (EFdA-TP) as substrate but, despite the presence of the 3′-hydroxyl group, the incorporated EFdA monophosphate (EFdA-MP) blocks further DNA synthesis since the enzyme is not able to efficiently translocate on a RNA:DNA or a DNA:DNA hybrid containing a 3′-terminal EFdA-MP [93] (Figure 10). In fact, on the one hand, the North (C2′-exo/C3′-endo) EFdA sugar ring conformation (which is the proper 3′-terminus position for in-line nucleophilic attack on the *α*-phosphate of the incoming dNTP) has been shown to be required for efficient binding at the primer-binding and RT polymerase active sites suggesting that, once incorporated into the DNA, the EFdA 3′-hydroxyl group is not likely to prevent by itself additional nucleotides incorporation, and, thus, it does not contribute to the mechanism of chain termination [94]. On the other hand, molecular modeling studies suggested that the 4′-ethynyl of EFdA may fit into a hydrophobic pocket defined by residues A114, Y215, F160, M184 and the aliphatic D185 chain [93]. Hence, it has been proposed that the presence of a 4′-ethynyl substitution on the ribose ring possibly hampers RT to translocate the 3′-EFdA-MP terminus DNA. Under these circumstances, RT is stabilized in a pretranslocation state which antagonizes the further nucleotide addition, since the dNTP-binding site is not accessible and the incorporation of the next complementary nucleotide is prevented [93]. Notably, in spite of the fact that the diminished translocation makes the 3′-EFdA-MP terminus DNA an excellent substrate for NRTI excision, the net excision process has been reported to be not very efficient, apparently because once the nucleotide is excised through pyrophosphorolysis to form EFdA-TP, the latter is rapidly reincorporated [93]. Moreover, it has been recently reported that EFdA is a poor substrate for DNA polymerase *γ* (it is incorporated 4,300-fold less than dATP), suggesting minimal mitochondrial toxicity [95].

#### 5.2.2. Delayed Chain Terminators RT Inhibitors

**Figure 11:**
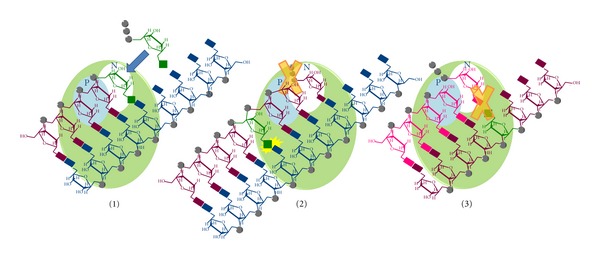
Mechanism of action of DCTRTIs. The RT is represented as a pale green circle with the priming binding site in cyan (P) and the nucleotide binding site in white (N). The RNA template is shown in blue and the (−)strand DNA in purple. DCTRTI triphosphate (strong green) is incorporated into the growing DNA chain (1). After further nucleotides addition, its presence blocks DNA elongation, probably through steric hindrance interference (yellow) between the RNA:DNA hybrid and the RT nucleic-acid-binding cleft (2). In addition, their incorporation can also block the synthesis of the (+)strand DNA affecting the base pairing (3).

 DCTRTIs are NRTIs that allow further incorporation of dNTPs into the growing DNA chain since they have a 3′-hydroxyl group. However, after further nucleotide addition, their presence blocks DNA elongation, probably through steric hindrance interference between the RNA:DNA hybrid and the RT nucleic acid binding cleft, close to the polymerase active site (Figure 11). They can also block the synthesis of the (+)strand DNA affecting the base pairing.

2′,3′-dideoxy-3′C-hydroxymethyl cytidine (PPI-801) (Figure 9) has been reported to allow the incorporation of one additional dNTP prior to mediating chain termination [65]. Interestingly, the incorporated PPI-801 is not accessible for nucleotide excision, and, therefore, this class of compounds is proposed to be attractive because it should be active also on NRTI-resistant strains with enhanced 3′-end nucleotide excision.

8-isopropyl-amino-2′-deoxyadenosine (8iPrNdA) (Figure 9) is a recently reported molecule belonging to a series of nucleoside analogs with a natural deoxyribose moiety and modifications at position 8 of the adenine base [96]. These modifications may induce a steric clash with helix *α*H in the thumb domain of the p66 subunit, causing delayed chain termination. In fact, once incorporated into the elongated DNA, 8iPrNdA stops the further DNA synthesis after the incorporation of three additional dNTPs [96]. Even though the potency and selectivity of 8iPrNdA are not very high, it is an interesting example of an NRTI with modifications on the adenine base and not on the sugar moiety.

#### 5.2.3. Lethal Mutagenesis RT Inhibitors

LMRTIs are NRTIs that allow further incorporation of dNTPs into the growing DNA chain. However, their incorporation causes a significant increase of nucleotide mismatches that determines a high mutation rate that eventually leads to viral replication suppression.

5-hydroxydeoxycytidine (5-OH-dC) (Figure 9) is a deoxycytidine analog that can efficiently base pair with both guanosine and adenosine nucleotides [97]. Viral growth in the presence of 5-OH-dC determines a 2.5-fold increase in G to A substitutions and a decline in viral infectivity over serial passages [97]. The fact that a relatively small increase in the HIV mutation frequency has a large effect on viral lethality substantiates the concept that the HIV mutation frequency is close to the error threshold for the viability of the quasispecies and that NRTIs that may significantly increase mutation frequency can act almost analogously to the cellular cytidine deaminase APOBEC3G [97].

5-aza-5,6-dihydro-2′-deoxycytidine (KP-1212) (Figure 9) is a deoxycytidine analog with a modified base and a natural sugar moiety that can also base pair with both guanosine and adenosine nucleotides [98]. The virus grown in the presence of KP-1212 accumulates a number of mutations that, eventually, stops its replication [98]. KP-1212 has been reported to interact also with DNA polymerase *γ* [99], suggesting a possible mitochondrial toxicity that, however, has not been observed in cells [98].

#### 5.2.4. Dinucleotide Tetraphosphates

As described above, nucleotides excision is a major mechanism of NRTI resistance. During this mechanism RT catalyzes the pyrophosphorolysis of, for instance, a 3′-AZT-MP terminated DNA. In fact, in the presence of the PPi donor ATP, RT catalyzes the excision reaction which results in the production of a dinucleoside tetraphosphate (i.i. A_p4_AZT) freeing the 3′-end for further DNA elongation. Notably, X-ray crystal studies have shown that the AMP part of the A_p4_AZT dinucleotide (Figure 9) binds differently to wt and drug-resistant mutant RTs [100]. These observations demonstrate that (i) RT can catalyze the reverse reaction and (ii) drug resistance mutations create a high-affinity ATP-binding site and open the possibility of designing drugs that can inhibit the enzyme mimicking the N_p4_N excision product that may be particularly active on NRTI-resistant strains. Up to now, a few N_p4_Ns have been synthesized that are able to inhibit wt and AZT-resistant RTs in the low micromolar range [101]. Notably, while the tetraphosphate linker, that avoids the intracellular phosphorylation step, is a potential advantage of these molecules, it is also an obstacle for their stability and cellular permeability. More studies dedicated to a further exploration of the ATP-binding site may lead to potent and innovative drugs.

## 6. New Nonnucleoside RT Inhibitors

**Figure 12:**
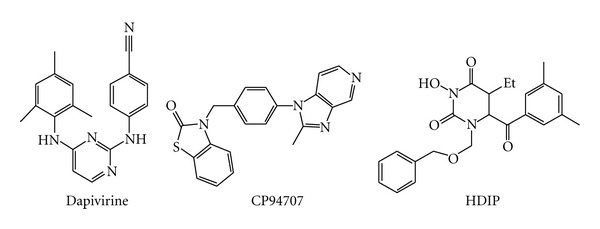
Chemical structures of new NNRTIs.

The NNRTIs therapeutic use is limited mainly by the selection of NNRTI resistant virus, even though drug hypersensitivity and/or serious central nervous system dysfunctions are also toxicity issues for some NNRTIs. For this reason, there is still an active focus on the development of new NNRTIs, especially for compounds with high potency against K103N, Y181C, and Y188V mutant viruses. Besides the fact that more than 30 different conformational classes of NNRTIs have been reported to date [102, 103], the recent development of new NNRTIs has been focused on the identification of molecules that retain high conformation flexibility and positional adaptability in order to adjust the inhibitor conformation to the NNRTI-binding pocket, whose shape is different according to the presence of the diverse amino acid residues involved in NNRTI resistance. In fact, while first-generation NNRTIs, such as nevirapine, delavirdine, or efavirenz, bind to RT in “two-wing” (or “butterfly-like”) conformation, the most recently developed NNRTIs show a “U” (or “horseshoe”) conformation which gives an increased plasticity to these derivatives [104, 105]. Success stories of such an approach are the latest approved NNRTIs, etravirine and rilpivirine (Figure 7), and another compound under clinical investigation in phase I/II clinical trials, dapivirine (Figure 12) [104, 105].

Another complementary strategy used to improve the NNRTIs performance is to design derivatives that make strong interactions with highly conserved amino acid residues in the NNRTI- binding pocket such as F227, W229, L234, and Y318 [105, 106]. In fact, these first three residues are part of the primer grip region that maintains the primer terminus in an appropriate orientation for the nucleophilic attack on the incoming dNTP. Specifically, the W229 residue is the prime candidate residue for drug design, and, in fact, among others, the above-mentioned rilpivirine has been reported to make strong interactions with the indole ring of W229.

Another reported interesting NNRTI is 3-(4-(2-methyl-1H-imidazo[4,5-c]pyridin-1-yl)benzyl)benzo[d]thiazol-2(3H)-one (CP94707) (Figure 12) that inhibits, even though not very potently, wt and mutant Y181C and Y188C RTs at the same concentrations and shows only a 2-fold reduction in potency of inhibition on K103N RT [107]. CP94707 makes little contact with Y181 and Y188 residues, while it makes aromatic ring stacking interactions with W229 amino acid [107]. In addition, CP94707 binding to RT results in rearrangement of the distally positioned Y115 side chain, 15 Å away, to a conformation that is incompatible with binding of dNTPs. Y115, in fact, can act as a gatekeeper residue that discriminates between deoxynucleotides and ribonucleotides. Therefore, it has been proposed that CP94707 may have a nonconventional mode of action [108].

An NNRTIs series of N-hydroxyimide derivatives, such as compound 1-((benzyloxy)methyl)-6-(3,5-dimethylbenzoyl)-5-ethyl-3-hydroxydihydropyrimidine-2,4(1H,3H)-dione (HDIP) (Figure 12), have been developed as dual RT and IN inhibitors (DRT-INI). In fact, they have been reported to inhibit both the RT-associated RDDP function and the IN activity [109, 110] and have been proposed to bind to the NNRTI-binding site and also chelate the magnesium ion in the IN active site [109, 110].

## 7. Nucleotide Competing RT Inhibitors

**Figure 13:**
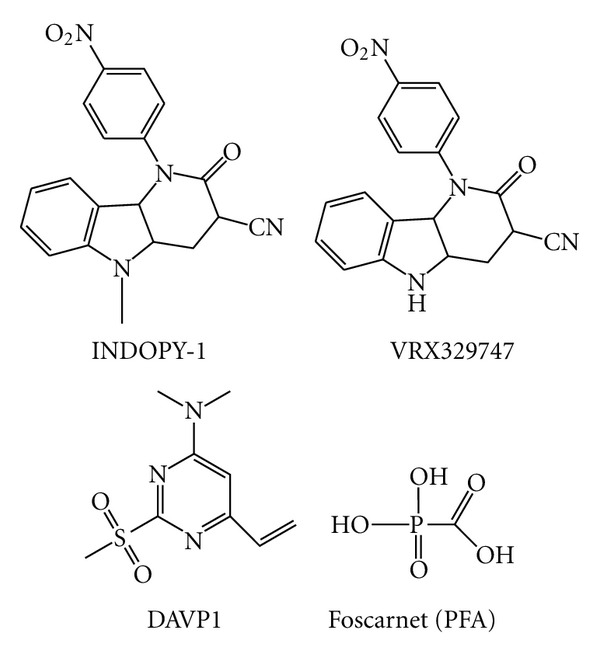
Chemical structures of NcRTIs.

A series of indolopyridones, therefore belonging to the NNRTIs, have been shown to inhibit RT interacting differently from the classic NNRTIs. In particular, 5-methyl-1-(4-nitrophenyl)-2-oxo-2,5-dihydro-1H pyrido[3,2-b]indole-3-carbonitrile (INDOPY-1) (Figure 13) (i) inhibits also HIV-2 RT [111], while the other NNRTIs are inactive against this enzyme; (ii) it is active against K103N, Y181C, and Y188C mutant RTs as potently as on wt RT, while it is 3.6-fold less active against the K103N/L100I double-mutant RT [112]; (iii) it is active on TAM viruses, while it is 3- to 8-fold less effective on M184V or Y115F mutant viruses, it is more than 100-fold less potent on the M184V/Y115F double-mutant virus, and it is slightly more effective on K65R mutant virus [111–113]. In addition, the INDOPY-1 analog 1-(4-nitrophenyl)-2-oxo-2,5-dihydro-1H-pyrido[3,2-b]indole-3-carbonitrile (VRX329747) (Figure 13) selected HIV-1 RT mutated at the amino acid residues M41L, A62V, S68N, G112S, V118I, and M184V, which are all located around the incoming nucleotide-binding site [112]. Further, binding and biochemical studies revealed that (i) the M184V mutation reduces the affinity to INDOPY-1, while the Y115F mutation facilitates the dNTP binding, and their combined effects enhance the ability of the enzyme to discriminate against the inhibitor [113]; (ii) RT complexed with INDOPY-1 is trapped in the posttranslocational state [113]; (iii) the INDOPY-1 has preference with respect to substrate primer identity since its binding to RT is higher on a DNA:DNA versus a RNA:DNA primer:template [114]; (iv) when assayed by steady-state kinetic analysis with homopolymeric template primers, INDOPY-1 inhibits RT-catalyzed DNA polymerization with a competitive [111] or mixed-type [112] mode with respect to dNTPs. Overall, these observations suggest that the binding site of the indolopyridones and nucleotide substrates can at least partially overlap and they are therefore proposed as Nucleotide competing RT inhibitors (NcRTIs). 

4-dimethylamino-6-vinylpyrimidines (DAVPs) is another class of compounds that have been reported to compete with the incoming dNTP and therefore can be considered NcRTI [115, 116]. However, differently from INDOPY-1, DAVP1 (Figure 13) is 4000- and 5000-fold less potent on mutant K103N and Y181C RTs, respectively [115], and binds also to unligated RT (while INDOPY-1 binds only to the RT:template:primer complex) [116]. X-ray crystal studies have confirmed that DAVP1 binds to an RT site that is distinct from the NNRTI-binding pocket, and it is close to the RT polymerase catalytic site [117]. This site is located in a hinge region, at the interface between the p66 thumb and p66 palm subdomains, that comprises the amino acid residues M230 and G231 (participating to the primer grip region and helping in the correct positioning of the 3′-OH end of the DNA primer), G262, K263 and W266 (involved in the template primer recognition), M184 and D186 (the first is involved in DNA synthesis fidelity, while the second is part of the catalytically essential YXDD motif) [117]. Hence, the DAVP1 binding site is located in a region critical for the correct positioning of the 3′-OH primer for the in-line nucleophilic attack by the incoming dNTP and the subsequent chemical bond formation with its *α*-phosphate. Notably, the X-ray study also revealed that in the RT/DAVP-1 complex the RT conformation is analogous to the “closed” conformation observed in unliganded RTs (with the p66 thumb subdomain folded into the DNA-binding cleft) and differs from that observed in RT/NNRTI complexes that has a hyperextended “open” conformation [117]. However, considering the proposed binding site, the reason for the loss of DAVP1 activity against K103N and Y181C mutant RTs remains unclear. While it has been hypothesized that DAVP1, owing to its small size, could travel between the NNRTI and nucleoside-binding pockets [117], more studies are needed to understand the DAVP1 mode of action.

## 8. PPi Analogs Inhibitors

Foscarnet (phosphonoformate, PFA) (Figure 13) is a PPi analogue that targets the DNA polymerase of herpes viruses as well as the RT of retroviruses [118]. Foscarnet is used intravenously to treat opportunistic viral infections, particularly CMV retinitis in patients with AIDS, but its pharmacokinetic profile is complicated by nephrotoxicity [119]. When assayed against HIV-1 RT, it competitively blocks pyrophosphorolysis and PPi exchange reactions, suggesting that foscarnet and PPi share overlapping binding sites [120]. It has been shown that foscarnet traps the RT pretranslocated complex preventing the binding of the next nucleotide, and, thus, the pretranslocated complex has been proposed as a target for drug discovery [121]. *In vivo* and *in vitro* foscarnet-resistant HIV-1 variants have been shown to carry mutations in the RT gene at several positions, including W88G/S, E89K/G, L92I, A114S, S156A, Q161L, and H208Y [122–125]. Notably, most of the mutations that reduce the susceptibility to PFA also confer hypersensitivity to AZT and it has been suggested that foscarnet analogs may inhibit the phosphorolytic rescue of NRTI-terminated primers and be used to prevent the excision-based mode of NRTI resistance [126].

## 9. RNase H Inhibitors

Despite the fact that the RT-associated RNase H function is essential for the reverse transcription process as well as the RT-associated DNA polymerase function, no effective RNase H RTIs (RHRTIs) have been developed yet. In the last few years, however, a few classes of RHRTI that are specifically targeted to the RNase H active site (Figure 4) have been identified [19, 127]. Most of them are able to chelate the divalent magnesium ion within the RNase H active site, but they also exert a high cellular toxicity, possibly due to an unspecific metal chelation, since the RNase H active site is an open pocket and offers, at least so far, little elements for selective small-molecule optimization.

### 9.1. Metal Chelating RHRTI

**Figure 14:**
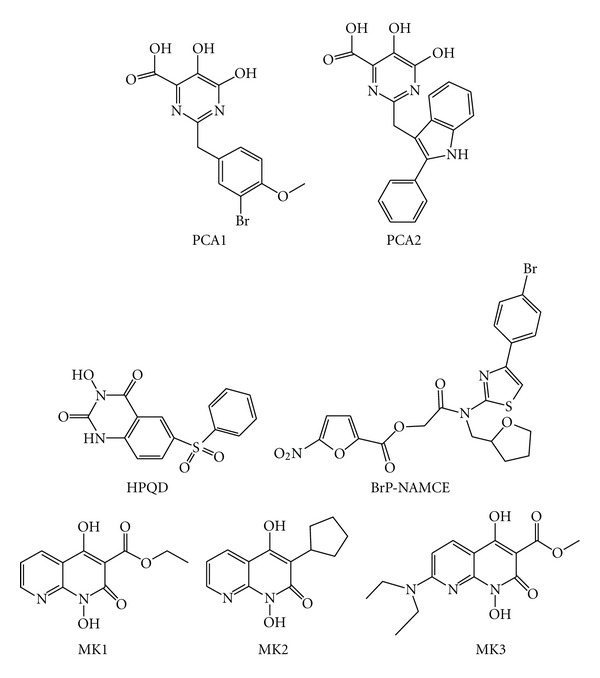
Chemical structures of metal chelating RHRTIs.

Pyrimidinol carboxylic acids 2-(3-bromo-4-methoxybenzyl)-5,6-dihydroxypyrimidine-4-carboxylic acid (PCA1), 5,6-dihydroxy-2-((2-phenyl-1H-indol-3-yl)methyl)pyrimidine-4-carboxylic acid (PCA2) and *N*-hydroxy quinazolinedione inhibitors 3-hydroxy-6-(phenylsulfonyl)quinazoline-2,4(1H,3H)-dione) (HPQD) (Figure 14) were designed to coordinate the two metal ions in the active site of RNase H and showed no interactions with the polymerase metal-binding site [128]. However, so far they have not been further developed.

Similarly, Nitrofuran-2-carboxylic acids derivatives such as the 5-nitro-furan-2-carboxylic acid [[4-(4-bromo-phenyl)-thiazol-2-yl]-(tetrahydro-furan-2-ylmethyl)-carbamoyl]-methyl ester (BrP-NAMCE) (Figure 14) were identified to inhibit the RNase H function by chelating the magnesium ion [129], and other analogs were also reported [130], but more derivatization studies are needed in order to develop effective inhibitors.

Naphthyridine derivatives ethyl 1,4-dihydroxy-2-oxo-1,2-dihydro-1,8-naphthyridine-3-carboxylate (MK1), 3-cyclopentyl-1,4-dihydroxy-1,8-naphthyridin-2(1H)-one (MK2) and methyl 7-(diethylamino)-1,4-dihydroxy-2-oxo-1,2-dihydro-1,8-naphthyridine-3-carboxylate (MK3) (Figure 14) have been reported to bind to the RNase H active site by coordinating the two metal ions, engaging the conserved catalytic DDE motif [131]. Interestingly, they were reported to be sandwiched by a loop containing residues A538 and H539 residues on the one side and N474 on the opposite side. In addition, MK3 was also shown to bind to a site adjacent to the NNRTI including amino acid residues L100, V108, Y181, Y183, D186, L187, K223, F227, L228, W229, and L234 [131]. Unlike the binding to the RNase H active site, the binding to this alternate site appears to be predominantly mediated via the hydrophobic interactions with the diethylaminophenoxy group unique to MK3. The rilevance of the MK3 binding to this site is not clear; however, the site is similar to the binding site for DHBNH (see later).

### 9.2. Dual RHRTI and IN Inhibitors

**Figure 15:**
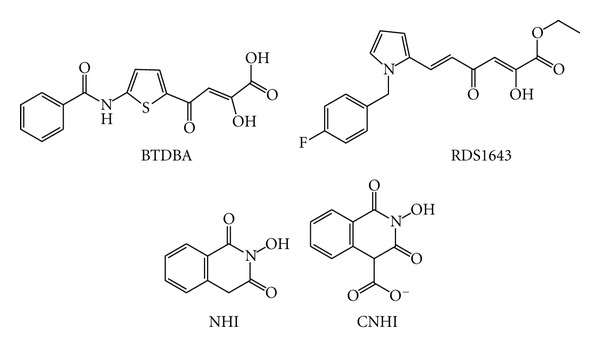
Chemical structures of dual RHRTI-INIs.

The first recently discovered RHRTIs were the diketo acid (DKA) derivatives 4-[5-(benzoylamino)thien-2-yl]-2,4-dioxobutanoic acid (BTDBA) (Figure 15) [132] and 6-[1-(4-fluorophenyl)methyl-1*H*pyrrol-2-yl)]-2,4-dioxo-5-hexenoic acid ethyl ester (RDS1643) (Figure 15) [133], that were independently developed against the HIV-1 IN. Due to similarities between RNase H and IN active sites, they were explored as RHRTIs and found to be active. Both of them are able to chelate Mg^2+^ in the RNase H catalytic site and are inactive on the DNA polymerase function [132, 133]. For this reason DKAs are currently under development as dual RNase H and INIs (DRH-INI) [19, 134–136].

Other derivatives that have also been developed as DRH-INIs are *N*-hydroxyimide. The prototype of these inhibitors was the 2-hydroxyisoquinoline-1,3(2*H*,4*H*)-diones (NHI) (Figure 15) [137, 138] that was shown, by crystal structures with the isolated RNase H domain, to bind to RT in a strictly metal dependent manner, confirming the metal-ion-mediated mode of action. More recently, other* N*-hydroxyimide derivatives were synthesized such as DRH-INIs [139, 140]. Interestingly, the methyl 2-Hydroxy-1,3-dioxo-1,2,3,4-tetrahydroisoquinoline-4-carboxylate analog (CNHI) (Figure 15) has also been shown to inhibit the replication of the double-mutant G140S/Q148H, which is the most resistant strain to the INI raltegravir [140], indicating that it is possible to design compounds with the same scaffold that may (i) inhibit both RNase H and IN and (ii) inhibit specifically one of the two enzymes. Further studies will be needed to dissect the specifics of the two active sites.

### 9.3. Nonmetal Chelator RHRTI

**Figure 16:**
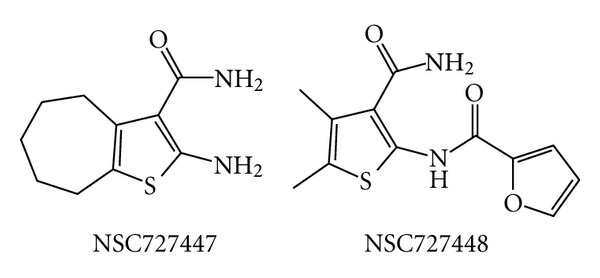
Chemical structures of nonmetal chelating RHRTIs.

Unlike the above-mentioned compounds, vinylogous ureas compounds 2-amino-5,6,7,8-tetrahydro-4*H*-cyclohepta[*b*]thiophene-3-carboxamide (NSC727447) and *N*-[3-(aminocarbonyl)-4,5-dimethyl-2-thienyl]-2-furancarboxamide (NSC727448) (Figure 16) that inhibit the RNase H function are ineffective on the DNA polymerase function, but they do not chelate the magnesium ion [141]. These two derivatives were further developed into more potent analogs that, however, were devoid of antiviral activity in cell culture [142]. Molecular modeling studies showed that they bind to an hydrophobic pocket comprising residues V276, C280, K281, K275, R277, and R284 of the p51 thumb and residues G541 and H539 of the RNase H domain (Figure 4) [142]. Further studies are certainly warranted since this new pocket is highly attractive for RHRTIs development.

## 10. Dual RNase H and Polymerase Inhibitors

**Figure 17:**
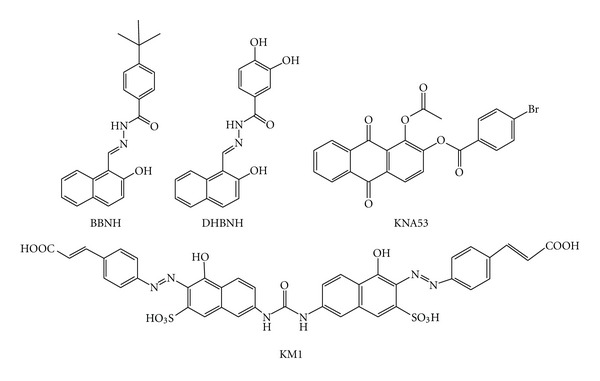
Chemical structures of dual RNase H and polymerase inhibitors.

An interesting class of RHRTIs is the hydrazone derivatives, whose first reported analog was *N*-(4-*tert*-butylbenzoyl)-2-hydroxy-1-naphthaldehyde hydrazone (BBNH) (Figure 17). Unlike other NNRTIs or RHRTIs, BBNH inhibits both the polymerase and the RNase H activities of HIV-1 RT [143] and therefore can be classified as dual NNRTI (DNNRTI). In addition, BBNH inhibits both RT-associated RNase H and RDDP activities of K103N, Y181I, Y188H, and Y188L mutant RTs with potency similar to wt RT, while, when assayed on Y181C mutant RT, it inhibits only the RDDP function and is inactive on the RNase H function [144]. This information, together with the data on other hydrazone derivatives that chelate the metal ion cofactor in the RNase H site [145], led to propose that two BBNH molecules could bind RT in two different sites, the first one in the polymerase domain, possibly near the NNRTI-binding site, and the second one possibly located in the RNase H domain. Subsequently, another derivative, (E)-3,4-dihydroxy-N′-((2-hydroxynaphthalen-1-yl)methylene)benzohydrazide (DHBNH) (Figure 17), has been reported to bind near the polymerase active site in a pocket different from the NNRTI-binding site and also >50 Å away from the RNase H active site (Figure 4) [146]. Hence, it was hypothesized that DHBNH may either perturb the trajectory of the template primer, so that RNase H cannot operate on its substrate, or that it may also bind to a second site, in or near the RNase H domain, that was not seen in the crystal. More recently, molecular docking studies on a series of hydrazone analogs proposed that they bind to a pocket that includes residues Y405, W406, Q500, and Y501 of p66 subunit, and, hence, they form hydrophobic interactions with RT and with base pairs in the groove of the RNA:DNA substrate [147]. In fact, residues D499 and A502, adjacent to Q500, which were perturbed by the hydrazone derivatives presence [147], are part of the primer grip of the RNase H domain and play a role in aligning the DNA:RNA substrate with the active site. Therefore, the hydrazones binding to Q500 may disrupt the primer grip's role in the activity of RNase H.

A second class of DNNRTI is a series of emodin [148] and alizarine anthraquinone derivatives [149, 150] such as 1-acetoxy-9,10-dioxo-9,10-dihydroanthracen-2-yl 4-bromobenzoate (KNA-53) (Figure 17), that inhibits both RT-associated functions of wt and K103N RTs and only the RNase H function of Y181C RT. Mode of action studies and molecular dynamic simulation led to proposing that the anthraquinone derivatives bind to the site adjacent to the NNRTI pocket, which was originally reported [146] for the hydrazones derivatives (Figure 4) [149]. Accordingly, it has been suggested that the anthraquinone inhibition of the RNase H function may be due to a change in the RNA:DNA hybrid RT accommodation, induced by their binding, which results in a possible variation in the nucleic acid trajectory toward the RNase H catalytic site [149].

A third class of DNNRTI is the naphthalenesulfonic acid derivatives that were originally reported to have a selective activity on the RT-associated RDDP function [151] and were further developed by structure-based design, molecular similarity, and combinatorial medicinal chemistry to obtain compound 2-Naphthalenesulfonic acid (4-hydroxy-7-[[[[5-hydroxy-6-[(4 cinnamylphenyl)azo]-7-sulfo-2-naphthalenyl]amino]-carbonyl]amino]-3-[(4-cinnamylphenyl)]azo (KM-1) (Figure 17), that inhibits both RT functions in the nanomolar range [152]. Subsequently, KM-1 was shown to weaken the RT DNA-binding affinity and to displace DNA from the enzyme [153]. Hence, it has been proposed to preclude the proper alignment of DNA at the polymerase active site, depleting the active DNA-bound RT complex required for nucleotide incorporation [153].

It is important to note that questions have been raised regarding the use of combinations between RHRTIs and NRTIs. In fact, RHRTIs have been proposed to lead to an increase in NRTIs resistance by mimicking the RNase H-dependent mechanism of NRTI resistance of some connection domain mutations [43]. Recently, however, studies on the effects of some RHRTIs on the HIV-1 susceptibility to AZT and 3TC have shown that none of the tested RHRTIs decreased NRTI susceptibility, while only one DNNRTI decreased AZT susceptibility by 5-fold [154]. More studies are needed to fully understand the interplay between RNase H inhibition and NRTIs susceptibility as well as its clinical relevance.

## 11. RT Dimerization Inhibitors

**Figure 18:**
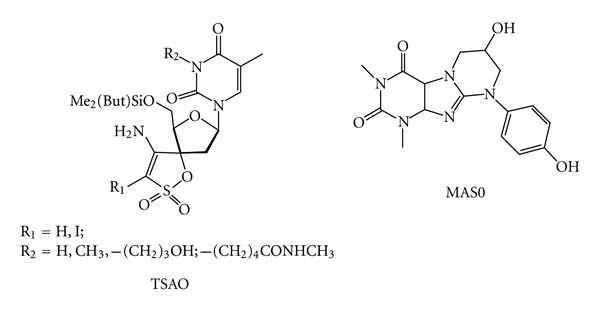
Chemical structures of DimRTIs.

RT dimerization is an absolute requirement for all enzymatic activities, and, accordingly, the development of inhibitors targeting the dimerization of RT represents a promising alternative antiviral strategy [155]. Up to now only a series of small molecules have been found which are able to inhibit RT dimerization. Among them are the above-mentioned BBNH derivative [143, 145] and the [2′,5′-bis-*O*-(*tert*-butyldimethylsilyl)-beta-D-ribofuranose]-3′-spiro-5′′-(4′′-amino-1′′,2′′-oxathiole-2′′,2′′-dioxide) (TSAO) (Figure 18) derivatives [156], that make extensive contact with the *β*7/*β*8 loop of the p51 subunit, that forms the “floor” of the NNRTI binding pocket and fits in a groove-like structure that constitutes the template:primer binding site in the p66 subunit. More recently, a structure-based ligand study has identified compounds 7-hydroxy-9-(4-hydroxyphenyl)-1,3-dimethyl-1,6,7,8,9,10a-hexahydropyrimido [2,1-f]purine-2,4(3H,4aH)-dione MAS0 as potent dimerization RT inhibitors (DimRTIs) (Figure 18) [157].

## 12. Other Potential Targets in RT

The increase in knowledge regarding HIV life cycle and specifically the function of the HIV RT and its essential interactions with other proteins will reveal potential drug targets. Even though no inhibitors have been identified yet, to the best of our knowledge, the DNA synthesis initiation (with an RNA:RNA primer), the PPT hydrolysis, the strand transfer, and pyrophosphorolysis RT functions are all potential aspects of the RT activities that may be targeted by small molecules. In addition, RT makes contact with other viral proteins such as NC and IN. These binding surfaces might be potential targets since their disruption may alter viral protein efficiency. Furthermore, some cellular factors have been described to interact with RT (and with the RT:IN complex) during reverse transcription and may have a role in its function [158]. Therefore, a better understanding of these interactions may offer other new target sites. Finally, intracellular immunity approaches may also involve proteins that affect RT functions and may thus offer additional target possibilities [31]. In conclusion, although RT has been the very first targeted HIV protein and is probably the most explored one, it still presents uninvestigated (or under investigation) functions and aspects that still make it a new fascinating target for innovative drug development.

## References

[1] Barré-Sinoussi F, Chermann JC, Rey F (1983). Isolation of a T-lymphotropic retrovirus from a patient at risk for acquired immune deficiency syndrome (AIDS). *Science*.

[2] Broder S, Gallo RC (1984). A pathogenic retrovirus (HTLV-III) linked to AIDS. *New England Journal of Medicine*.

[3] Mehellou Y, De Clercq E (2010). Twenty-six years of anti-HIV drug discovery: where do we stand and where do we go?. *Journal of Medicinal Chemistry*.

[4] Tsibris AMN, Hirsch MS (2010). Antiretroviral therapy in the clinic. *Journal of Virology*.

[5] Ratner L, Haseltine W, Patarca R (1985). Complete nucleotide sequence of the AIDS virus, HTLV-III. *Nature*.

[6] Hughes SH, Arnold E, Hostomsky Z, Crouch RJ, Toulmé JJ (1998). RNase H of retroviral reverse transcriptases. *Ribonucleases H*.

[7] Huber HE, Richardson CC (1990). Processing of the primer for plus strand DNA synthesis by human immunodeficiency virus 1 reverse transcriptase. *Journal of Biological Chemistry*.

[8] Rausch JW, Le Grice SFJ (2004). ’Binding, bending and bonding’: polypurine tract-primed initiation of plus-strand DNA synthesis in human immunodeficiency virus. *International Journal of Biochemistry and Cell Biology*.

[9] Basu VP, Song M, Gao L, Rigby ST, Hanson MN, Bambara RA (2008). Strand transfer events during HIV-1 reverse transcription. *Virus Research*.

[10] Divita G, Rittinger K, Geourjon C, Deleage G, Goody RS (1995). Dimerization kinetics of HIV-1 and HIV-2 reverse transcriptase: a two step process. *Journal of Molecular Biology*.

[11] Kohlstaedt LA, Wang J, Friedman JM, Rice PA, Steitz TA (1992). Crystal structure at 3.5 A resolution of HIV-1 reverse transcriptase complexes with an inhibitor. *Science*.

[12] Jacobo-Molina A, Ding J, Nanni RG (1993). Crystal structure of human immunodeficiency virus type 1 reverse transcriptase complexed with double-stranded DNA at 3.0 A resolution shows bent DNA. *Proceedings of the National Academy of Sciences of the United States of America*.

[13] Liu S, Abbondanzieri EA, Rausch JW, Le Grice SFJ, Zhuang X (2008). Slide into action: dynamic shuttling of HIV reverse transcriptase on nucleic acid substrates. *Science*.

[14] Abbondanzieri EA, Bokinsky G, Rausch JW, Zhang JX, Le Grice SFJ, Zhuang X (2008). Dynamic binding orientations direct activity of HIV reverse transcriptase. *Nature*.

[15] Steltz TA (1998). A mechanism for all polymerases. *Nature*.

[16] Ghosh M, Jacques PS, Rodgers DW, Ottman M, Darlix JL, Le Grice SFJ (1996). Alterations to the primer grip of p66 HIV-1 reverse transcriptase and their consequences for template-primer utilization. *Biochemistry*.

[17] Sarafianos SG, Marchand B, Das K (2009). Structure and function of HIV-1 reverse transcriptase: molecular mechanisms of polymerization and inhibition. *Journal of Molecular Biology*.

[18] Kellinger MW, Johnson KA (2010). Nucleotide-dependent conformational change governs specificity and analog discrimination by HIV reverse transcriptase. *Proceedings of the National Academy of Sciences of the United States of America*.

[19] Tramontano E, Di Santo R (2010). HIV-1 RT-associated Rnase H function inhibitors: recent advances in drug development. *Current Medicinal Chemistry*.

[20] Nowotny M, Gaidamakov SA, Crouch RJ, Yang W (2005). Crystal structures of RNase H bound to an RNA/DNA hybrid: substrate specificity and metal-dependent catalysis. *Cell*.

[21] Rosta E, Nowotny M, Yang W, Hummer G (2011). Catalytic mechanism of RNA backbone cleavage by ribonuclease H from quantum mechanics/molecular mechanics simulations. *Journal of the American Chemical Society*.

[22] Mizrahi V, Usdin MT, Harington A, Dudding LR (1990). Site-directed mutagenesis of the conserved Asp-443 and Asp-498 carboxy-terminal residues of HIV-1 reverse transcriptase. *Nucleic Acids Research*.

[23] Mizrahi V, Brooksbank RL, Nkabinde NC (1994). Mutagenesis of the conserved aspartic acid 443, glutamic acid 478, asparagine 494, and aspartic acid 498 residues in the ribonuclease H domain of p66/p51 human immunodeficiency virus type I reverse transcriptase. Expression and biochemical analysis. *Journal of Biological Chemistry*.

[24] Beilhartz GL, Götte M (2010). HIV-1 ribonuclease H: structure, catalytic mechanism and inhibitors. *Viruses*.

[25] Sarafianos SG, Das K, Tantillo C (2001). Crystal structure of HIV-1 reverse transcriptase in complex with a polypurine tract RNA:DNA. *EMBO Journal*.

[26] Champoux JJ, Schultz SJ (2009). Ribonuclease H: properties, substrate specificity and roles in retroviral reverse transcription. *FEBS Journal*.

[27] Furfine ES, Reardon JE (1991). Reverse transcriptase. RNase H from the human immunodeficiency virus. Relationship of the DNA polymerase and RNA hydrolysis activities. *Journal of Biological Chemistry*.

[28] Telesnitsky A, Goff SP, Coffin JM, Hughes SH, Varmus HE (1997). Reverse Transcriptase and the generation of retroviral DNA. *Retroviruses*.

[29] Ji X, Klarmann GJ, Preston BD (1996). Effect of human immunodeficiency virus type 1 (HIV-1) nucleocapsid protein on HIV-1 reverse transcriptase activity in vitro. *Biochemistry*.

[30] Grohmann D, Godet J, Mély Y, Darlix JL, Restle T (2008). HIV-1 nucleocapsid traps reverse transcriptase on nucleic acid substrates. *Biochemistry*.

[31] Aguiar RS, Peterlin BM (2008). APOBEC3 proteins and reverse transcription. *Virus Research*.

[32] Arion D, Kaushik N, McCormick S, Borkow G, Parniak MA (1998). Phenotypic mechanism of HIV-1 resistance to 3′-azido-3′-deoxythymidine (AZT): increased polymerization processivity and enhanced sensitivity to pyrophosphate of the mutant viral reverse transcriptase. *Biochemistry*.

[33] Meyer PR, Matsuura SE, So AG, Scott WA (1998). Unblocking of chain-terminated primer by HIV-1 reverse transcriptase through a nucleotide-dependent mechanism. *Proceedings of the National Academy of Sciences of the United States of America*.

[34] Meyer PR, Matsuura SE, Mohsin Mian A, So AG, Scott WA (1999). A mechanism of AZT resistance: an increase in nucleotide-dependent primer unblocking by mutant HIV-1 reverse transcriptase. *Molecular Cell*.

[35] Simpson DM, Tagliati M (1995). Nucleoside analogue-associated peripheral neuropathy in human immunodeficiency virus infection. *Journal of Acquired Immune Deficiency Syndromes and Human Retrovirology*.

[36] Schinazi RF, Lloyd RM, Nguyen MH (1993). Characterization of human immunodeficiency viruses resistant to oxathiolane-cytosine nucleosides. *Antimicrobial Agents and Chemotherapy*.

[37] Schuurman R, Nijhuis M, Van Leeuwen R (1995). Rapid changes in human immunodeficiency virus type 1 RNA load and appearance of drug-resistant virus populations in persons treated with lamivudine (3TC). *Journal of Infectious Diseases*.

[38] Sarafianos SG, Das K, Clark AD (1999). Lamivudine (3TC) resistance in HIV-1 reverse transcriptase involves steric hindrance with *β*-branched amino acids. *Proceedings of the National Academy of Sciences of the United States of America*.

[39] Huang H, Chopra R, Verdine GL, Harrison SC (1998). Structure of a covalently trapped catalytic complex of HIV-1 reverse transcriptase: implications for drug resistance. *Science*.

[40] Boyer PL, Sarafianos SG, Arnold E, Hughes SH (2001). Selective excision of AZTMP by drug-resistant human immunodeficiency virus reverse transcriptase. *Journal of Virology*.

[41] Dharmasena S, Pongracz Z, Arnold E, Sarafianos SG, Parniak MA (2007). 3′-azido-3′-deoxythymidine-(5′)-tetraphospho-(5′)-adenosine, the product of ATP-mediated excision of chain-terminating AZTMP, is a potent chain-terminating substrate for HIV-1 reverse transcriptase. *Biochemistry*.

[42] Yahi N, Tamalet C, Tourrès C, Tivoli N, Fantini J (2000). Mutation L210W of HIV-1 reverse transcriptase in patients receiving combination therapy: incidence, association with other mutations, and effects on the structure of mutated reverse transcriptase. *Journal of Biomedical Science*.

[43] Nikolenko GN, Delviks-Frankenberry KA, Palmer S (2007). Mutations in the connection domain of HIV-1 reverse transcriptase increase 3′-azido-3′-deoxythymidine resistance. *Proceedings of the National Academy of Sciences of the United States of America*.

[44] Yap SH, Sheen CW, Fahey J (2007). N348I in the connection domain of HIV-1 reverse transcriptase confers zidovudine and nevirapine resistance. *PLoS Medicine*.

[45] Delviks-Frankenberry KA, Nikolenko GN, Barr R, Pathak VK (2007). Mutations in human immunodeficiency virus type 1 RNase H primer grip enhance 3′-azido-3′-deoxythymidine resistance. *Journal of Virology*.

[46] Brehm JH, Koontz D, Meteer JD, Pathak V, Sluis-Cremer N, Mellors JW (2007). Selection of mutations in the connection and RNase H domains of human immunodeficiency virus type 1 reverse transcriptase that increase resistance to 3′-azido-3′-dideoxythymidine. *Journal of Virology*.

[47] Hachiya A, Kodama EN, Sarafianos SG (2008). Amino acid mutation N348I in the connection subdomain of human immunodeficiency virus type 1 reverse transcriptase confers multiclass resistance to nucleoside and nonnucleoside reverse transcriptase inhibitors. *Journal of Virology*.

[48] Zelina S, Sheen CW, Radzio J, Mellors JW, Sluis-Cremer N (2008). Mechanisms by which the G333D mutation in human immunodeficiency virus type 1 reverse transcriptase facilitates dual resistance to zidovudine and lamivudine. *Antimicrobial Agents and Chemotherapy*.

[49] Delviks-Frankenberry KA, Nikolenko GN, Pathak VK (2010). The “connection” between HIV drug resistance and RNase H. *Viruses*.

[50] Balzarini J, Naesens L, Aquaro S (1999). Intracellular metabolism of CycloSaligenyl 3′azido-2′,3′- dideoxythymidine monophosphate, a prodrug of 3′-azido-2′,3′-dideoxythymidine (zidovudine). *Molecular Pharmacology*.

[51] Squires KE (2001). An introduction to nucleoside and nucleotide analogues. *Antiviral Therapy*.

[52] Das K, Martinez SE, Bauman JD, Arnold E (2012). HIV-1 reverse transcriptase complex with DNA and nevirapine reveals non-nucleoside inhibition mechanism. *Nature Structural & Molecular Biology*.

[53] Mui PW, Jacober SP, Hargrave KD, Adams J (1992). Crystal structure of nevirapine, a non-nucleoside inhibitor of HIV-1 reverse transcriptase, and computational alignment with a structurally diverse inhibitor. *Journal of Medicinal Chemistry*.

[54] Schäfer W, Friebe WG, Leinert H (1993). Non-nucleoside inhibitors of HIV-1 reverse transcriptase: molecular modeling and X-ray structure investigations. *Journal of Medicinal Chemistry*.

[55] Ding J, Das K, Tantillo C (1995). Structure of HIV-1 reverse transcriptase in a complex with the non-nucleoside inhibitor *α*-APA R 95845 at 2.8 A resolution. *Structure*.

[56] Mager PP (2003). Hybrid canonical-correlation neural-network approach applied to nonnucleoside HIV-1 reverse transcriptase inhibitors (HEPT derivatives). *Current Medicinal Chemistry*.

[57] Sluis-Cremer N, Temiz NA, Bahar I (2004). Conformational changes in HIV-1 reverse transcriptase induced by nonnucleoside reverse transcriptase inhibitor binding. *Current HIV Research*.

[58] De Clercq E (1999). Perspectives of non-nucleoside reverse transcriptase inhibitors (NNRTIs) in the therapy of HIV-1 infection. *Farmaco*.

[59] Balzarini J (2004). Current status of the non-nucleoside reverse transcriptase inhibitors of human immunodeficiency virus type 1. *Current Topics in Medicinal Chemistry*.

[60] Esnouf RM, Ren J, Hopkins AL (1997). Unique features in the structure of the complex between HIV-1 reverse transcriptase and the bis(heteroaryl)piperazine (BHAP) U-90152 explain resistance mutations for this nonnucleoside inhibitor. *Proceedings of the National Academy of Sciences of the United States of America*.

[61] Ren J, Stammers DK (2008). Structural basis for drug resistance mechanisms for non-nucleoside inhibitors of HIV reverse transcriptase. *Virus Research*.

[62] Mellors JW, Dutschman GE, Im GJ, Tramontano E, Winkler SR, Cheng YC (1992). In vitro selection and molecular characterization of human immunodeficiency virus-1 resistant to non-nucleoside inhibitors of reverse transcriptase. *Molecular Pharmacology*.

[63] Mellors JW, Im GJ, Tramontano E (1993). A single conservative amino acid substitution in the reverse transcriptase of human immunodeficiency virus-1 confers resistance to (+)-(5S)-4,5,6,7- tetrahydro-5-methyl-6-(3-methyl-2-butenyl)imidazo[4,5,1- jk][1,4]benzodiazepin-2(1H)-thione (TIBO R82150). *Molecular Pharmacology*.

[64] Nikolenko GN, Delviks-Frankenberry KA, Pathak VK (2010). A novel molecular mechanism of dual resistance to nucleoside and nonnucleoside reverse transcriptase inhibitors. *Journal of Virology*.

[65] Cihlar T, Ray AS (2010). Nucleoside and nucleotide HIV reverse transcriptase inhibitors: 25 years after zidovudine. *Antiviral Research*.

[66] Ho HT, Hitchcock MJ (1989). Cellular pharmacology of 2′,3′-dideoxy-2′,3′-didehydrothymidine, a nucleoside analog active against human immunodeficiency virus. *Antimicrobial Agents and Chemotherapy*.

[67] Waters LJ, Moyle G, Bonora S (2007). Abacavir plasma pharmacokinetics in the absence and presence of atazanavir/ritonavir or lopinavir/ritonavir and vice versa in HIV-infected patients. *Antiviral Therapy*.

[68] Mcdowell JA, Chittick GE, Stevens CP, Edwards KD, Stein DS (2000). Pharmacokinetic interaction of abacavir (1592U89) and ethanol in human immunodeficiency virus-infected adults. *Antimicrobial Agents and Chemotherapy*.

[69] Johnson JA, Li JF, Wei X (2008). Minority HIV-1 drug resistance mutations are present in antiretroviral treatment-naive populations and associate with reduced treatment efficacy. *PLoS Medicine*.

[70] Bethell RC, Lie YS, Parkin NT (2005). In vitro activity of SPD754, a new deoxycytidine nucleoside reverse transcriptase inhibitor (NRTI), against 215 HIV-1 isolates resistant to other NRTIs. *Antiviral Chemistry and Chemotherapy*.

[71] Gu Z, Allard B, De Muys JM (2006). In vitro antiretroviral activity and in vitro toxicity profile of SPD754, a new deoxycytidine nucleoside reverse transcriptase inhibitor for treatment of human immunodeficiency virus infection. *Antimicrobial Agents and Chemotherapy*.

[72] Cox S, Southby J (2009). Apricitabine—a novel nucleoside reverse transcriptase inhibitor for the treatment of HIV infection that is refractory to existing drugs. *Expert Opinion on Investigational Drugs*.

[73] de Baar MP, de Rooij ER, Smolders KGM, van Schijndel HB, Timmermans EC, Bethell R (2007). Effects of apricitabine and other nucleoside reverse transcriptase inhibitors on replication of mitochondrial DNA in HepG2 cells. *Antiviral Research*.

[74] Bethell R, De Muys J, Lippens J (2007). In vitro interactions between apricitabine and other deoxycytidine analogues. *Antimicrobial Agents and Chemotherapy*.

[75] Lin TS, Luo MZ, Liu MC (1996). Design and synthesis of 2′,3′-dideoxy-2′,3′-didehydro-*β*-L-cytidine (*β*- L-d4C) and 2′,3’-dideoxy-2′-3′-didehydro-*β*-L-5-fluorocytidine (*β*-L-Fd4C), two exceptionally potent inhibitors of human hepatitis B virus (HBV) and potent inhibitors of human immunodeficiency virus (HIV) in vitro. *Journal of Medicinal Chemistry*.

[76] Dutschman GE, Bridges EG, Liu SH (1998). Metabolism of 2′,3′-dideoxy-2′,3′-didehydro-*β*-L(-)-5-fluorocytidine and its activity in combination with clinically approved anti-human immunodeficiency virus *β*-D(+) nucleoside analogs in vitro. *Antimicrobial Agents and Chemotherapy*.

[77] Hammond JL, Parikh UM, Koontz DL (2005). In vitro selection and analysis of human immunodeficiency virus type 1 resistant to derivatives of *β*-2′,3′-didehydro-2′,3′-dideoxy-5-fluorocytidine. *Antimicrobial Agents and Chemotherapy*.

[78] Parikh UM, Koontz DL, Chu CK, Schinazi RF, Mellors JW (2005). In vitro activity of structurally diverse nucleoside analogs against human immunodeficiency virus type 1 with the K65R mutation in reverse transcriptase. *Antimicrobial Agents and Chemotherapy*.

[79] Gu Z, Wainberg MA, Nguyen-Ba N (1999). Mechanism of action and in vitro activity of 1′,3′-dioxolanylpurine nucleoside analogues against sensitive and drug-resistant human immunodeficiency virus type 1 variants. *Antimicrobial Agents and Chemotherapy*.

[80] Mewshaw JP, Myrick FT, Wakefield DACS (2002). Dioxolane guanosine, the active form of the prodrug diaminopurine dioxolane, is a potent inhibitor of drug-resistant HIV-1 isolates from patients for whom standard nucleoside therapy fails. *Journal of Acquired Immune Deficiency Syndromes*.

[81] Bazmi HZ, Hammond JL, Cavalcanti SCH, Chu CK, Schinazi RF, Mellors JW (2000). In vitro selection of mutations in the human immunodeficiency virus type 1 reverse transcriptase that decrease susceptibility to (-)-*β*-D-dioxolane- guanosine and suppress resistance to 3′-azido-3′-deoxythymidine. *Antimicrobial Agents and Chemotherapy*.

[82] Furman PA, Jeffrey J, Kiefer LL (2001). Mechanism of action of 1-*β*-D-2,6-diaminopurine dioxolane, a prodrug of the human immunodeficiency virus type 1 inhibitor 1-*β*-D-dioxolane guanosine. *Antimicrobial Agents and Chemotherapy*.

[83] Feng JY, Anderson KS (1999). Mechanistic studies comparing the incorporation of (+) and (-) isomers of 3TCTP by HIV-1 reverse transcriptase. *Biochemistry*.

[84] Shewach DS, Liotta DC, Schinazi RF (1993). Affinity of the antiviral enantiomers of oxathiolane cytosine nucleosides for human 2′-deoxycytidine kinase. *Biochemical Pharmacology*.

[85] Schinazi RF, McMillan A, Cannon D (1992). Selective inhibition of human immunodeficiency viruses by racemates and enantiomers of cis-5-fluoro-1-[2-(hydroxymethyl)-1,3-oxathiolan-5-yl]cytosine. *Antimicrobial Agents and Chemotherapy*.

[86] Schinazi RF, McMillan A, Lloyd RL, Schlueter-Wirtz S, Liotta DC, Chu CK (1997). Molecular properties of HIV-1 resistant to (+)-enantiomers and racemates of oxathiolane cytosine nucleosides and their potential for the treatment of HIV and HBV infections. *Antiviral Research*.

[87] Herzmann C, Arastèh K, Murphy RL (2005). Safety, pharmacokinetics, and efficacy of (+/-)-*β*-2′,3′- dideoxy-5-fluoro-3′-thiacytidine with efavirenz and stavudine in antiretroviral-naive human immunodeficiency virus-infected patients. *Antimicrobial Agents and Chemotherapy*.

[88] Feng JY, Murakami E, Zorca SM (2004). Relationship between antiviral activity and host toxicity: comparison of the incorporation efficiencies of 2′,3′-Dideoxy-5-Fluoro-3′ -Thiacytidine-triphosphate analogs by human immunodeficiency virus type 1 reverse transcriptase and human mitochondrial DNA polymerase. *Antimicrobial Agents and Chemotherapy*.

[89] Dutschman GE, Grill SP, Gullen EA (2004). Novel 4′-substituted stavudine analog with improved anti-human immunodeficiency virus activity and decreased cytotoxicity. *Antimicrobial Agents and Chemotherapy*.

[90] Herdewijn P, Balzarini J, Baba M (1988). Synthesis and anti-HIV activity of different sugar-modified pyrimidine and purine nucleosides. *Journal of Medicinal Chemistry*.

[91] Hayakawa H, Kohgo S, Kitano K (2004). Potential of 4′-C-substituted nucleosides for the treatment of HIV-1. *Antiviral Chemistry and Chemotherapy*.

[92] Kawamoto A, Kodama E, Sarafianos SG (2008). 2′-Deoxy-4′-C-ethynyl-2-halo-adenosines active against drug-resistant human immunodeficiency virus type 1 variants. *International Journal of Biochemistry and Cell Biology*.

[93] Michailidis E, Marchand B, Kodama EN (2009). Mechanism of inhibition of HIV-1 reverse transcriptase by 4′-ethynyl-2-fluoro-2′-deoxyadenosine triphosphate, a translocation-defective reverse transcriptase inhibitor. *Journal of Biological Chemistry*.

[94] Kirby KA, Singh K, Michailidis E (2011). The sugar ring conformation of 4′-ethynyl-2-fluoro-2′-deoxyadenosine and its recognition by the polymerase active site of HIV reverse transcriptase. *Cellular and Molecular Biology*.

[95] Sohl CD, Singh K, Kasiviswanathan R (2012). The mechanism of interaction of human mitochondrial DNA *γ* with the novel nucleoside reverse transcriptase inhibitor 4′-Ethynyl-2-Fluoro-2′-deoxyadenosine indicates a low potential for host toxicity. *Antimicrobial Agents and Chemotherapy*.

[96] Vivet?Boudou V, Isel C, Sleiman M (2011). 8-modified-2′-deoxyadenosine analogues induce delayed polymerization arrest during HIV-1 reverse transcription. *PLoS ONE*.

[97] Loeb LA, Essigmann JM, Kazazi F, Zhang J, Rose KD, Mullins JI (1999). Lethal mutagenesis of HIV with mutagenic nucleoside analogs. *Proceedings of the National Academy of Sciences of the United States of America*.

[98] Harris KS, Brabant W, Styrchak S, Gall A, Daifuku R (2005). KP-1212/1461, a nucleoside designed for the treatment of HIV by viral mutagenesis. *Antiviral Research*.

[99] Smith RA, Loeb LA, Preston BD (2005). Lethal mutagenesis of HIV. *Virus Research*.

[100] Tu X, Das K, Han Q (2010). Structural basis of HIV-1 resistance to AZT by excision. *Nature Structural and Molecular Biology*.

[101] Meyer PR, Smith AJ, Matsuura SE, Scott WA (2006). Chain-terminating dinucleoside tetraphosphates are substrates for DNA polymerization by human immunodeficiency virus type 1 reverse transcriptase with increased activity against thymidine analogue-resistant mutants. *Antimicrobial Agents and Chemotherapy*.

[102] Sluis-Cremer N, Tachedjian G (2008). Mechanisms of inhibition of HIV replication by non-nucleoside reverse transcriptase inhibitors. *Virus Research*.

[103] Zhan P, Chen X, Li D, Fang Z, De Clercq E, Liu X HIV-1 NNRTIs: structural diversity, pharmacophore similarity, and implications for drug design.

[104] Das K, Lewi PJ, Hughes SH, Arnold E (2005). Crystallography and the design of anti-AIDS drugs: conformational flexibility and positional adaptability are important in the design of non-nucleoside HIV-1 reverse transcriptase inhibitors. *Progress in Biophysics and Molecular Biology*.

[105] Zhan P, Liu X, Li Z, Pannecouque C, De Clercq E (2009). Design strategies of novel NNRTIs to overcome drug resistance. *Current Medicinal Chemistry*.

[106] Janssen PAJ, Lewi PJ, Arnold E (2005). In search of a novel anti-HIV drug: multidisciplinary coordination in the discovery of 4-[[4-[[4-[(1E)-2-cyanoethenyl]-2,6-dimethylphenyl]amino]-2- pyrimidinyl]amino]benzonitrile (R278474, rilpivirine). *Journal of Medicinal Chemistry*.

[107] Pata JD, Stirtan WG, Goldstein SW, Steitz TA (2004). Structure of HIV-1 reverse transcriptase bound to an inhibitor active against mutant reverse transcriptases resistant to other nonnucleoside inhibitors. *Proceedings of the National Academy of Sciences of the United States of America*.

[108] Ren J, Stammers DK (2005). HIV reverse transcriptase structures: designing new inhibitors and understanding mechanisms of drug resistance. *Trends in Pharmacological Sciences*.

[109] Tang J, Maddali K, Dreis CD (2011). N-3 hydroxylation of pyrimidine-2,4-diones yields dual inhibitors of HIV reverse transcriptase and integrase. *ACS Medicinal Chemistry Letters*.

[110] Tang J, Maddali K, Dreis CD (2011). 6-Benzoyl-3-hydroxypyrimidine-2,4-diones as dual inhibitors of HIV reverse transcriptase and integrase. *Bioorganic and Medicinal Chemistry Letters*.

[111] Jochmans D, Deval J, Kesteleyn B (2006). Indolopyridones inhibit human immunodeficiency virus reverse transcriptase with a novel mechanism of action. *Journal of Virology*.

[112] Zhang Z, Walker M, Xu W (2006). Novel nonnucleoside inhibitors that select nucleoside inhibitor resistance mutations in human immunodeficiency virus type 1 reverse transcriptase. *Antimicrobial Agents and Chemotherapy*.

[113] Ehteshami M, Scarth BJ, Tchesnokov EP (2008). Mutations M184V and Y115F in HIV-1 reverse transcriptase discriminate against “nucleotide-competing reverse transcriptase inhibitors”. *Journal of Biological Chemistry*.

[114] Auger A, Beilhartz GL, Zhu S (2011). Impact of primer-induced conformational dynamics of HIV-1 reverse transcriptase on polymerase translocation and inhibition. *The Journal of Biological Chemistry*.

[115] Maga G, Radi M, Zanoli S (2007). Discovery of non-nucleoside inhibitors of HIV-1 reverse transcriptase competing with the nucleotide substrate. *Angewandte Chemie*.

[116] Radi M, Falciani C, Contemori L (2008). A multidisciplinary approach for the identification of novel HIV-1 non-nucleoside reverse transcriptase inhibitors: S-DABOCs and DAVPs. *ChemMedChem*.

[117] Freisz S, Bec G, Radi M (2010). Crystal structure of HIV-1 reverse transcriptase bound to a non-nucleoside inhibitor with a novel mechanism of action. *Angewandte Chemie*.

[118] Oberg B (1989). Antiviral effects of phosphonoformate (PFA, foscarnet sodium). *Pharmacology and Therapeutics*.

[119] Razonable RR (2011). Antiviral drugs for viruses other than human immunodeficiency virus. *Mayo Clinic Proceedings*.

[120] Derse D, Bastow KF, Cheng Y (1982). Characterization of the DNA polymerases induced by a group of herpes simplex virus type I variants selected for growth in the presence of phosphonoformic acid. *Journal of Biological Chemistry*.

[121] Marchand B, Tchesnokov EP, Götte M (2007). The pyrophosphate analogue foscarnet traps the pre-translocational state of HIV-1 reverse transcriptase in a Brownian ratchet model of polymerase translocation. *Journal of Biological Chemistry*.

[122] Mellors JW, Bazmi HZ, Schinazi RF (1995). Novel mutations in reverse transcriptase of human immunodeficiency virus type 1 reduce susceptibility to foscarnet in laboratory and clinical isolates. *Antimicrobial Agents and Chemotherapy*.

[123] Tachedjian G, Hooker DJ, Gurusinghe AD (1995). Characterisation of foscarnet-resistant strains of human immunodeficiency virus type 1. *Virology*.

[124] Im GJ, Tramontano E, Gonzalez CJ, Cheng YC (1993). Identification of the amino acid in the human immunodeficiency virus type 1 reverse transcriptase involved in the pyrophosphate binding of antiviral nucleoside triphosphate analogs and phosphonoformate. Implications for multiple drug resistance. *Biochemical Pharmacology*.

[125] Tramontano E, Piras G, Mellors JW, Putzolu M, Bazmi HZ, La Colla P (1998). Biochemical characterization of HIV-1 reverse transcriptases encoding mutations at amino acid residues 161 and 208 involved in resistance to phosphonoformate. *Biochemical Pharmacology*.

[126] Cruchaga C, Ansó E, Rouzaut A, Martínez-Irujo JJ (2006). Selective excision of chain-terminating nucleotides by HIV-1 reverse transcriptase with phosphonoformate as substrate. *Journal of Biological Chemistry*.

[127] Tramontano E (2006). HIV-1 RNase H: recent progress in an exciting, yet little explored, drug target. *Mini-Reviews in Medicinal Chemistry*.

[128] Lansdon EB, Liu Q, Leavitt SA (2011). Structural and binding analysis of pyrimidinol carboxylic acid and N-hydroxy quinazolinedione HIV-1 RNase H inhibitors. *Antimicrobial Agents and Chemotherapy*.

[129] Fuji H, Urano E, Futahashi Y (2009). Derivatives of 5-nitro-furan-2-carboxylic acid carbamoylmethyl ester inhibit RNase H activity associated with HIV-1 reverse transcriptase. *Journal of Medicinal Chemistry*.

[130] Yanagita H, Urano E, Matsumoto K (2011). Structural and biochemical study on the inhibitory activity of derivatives of 5-nitro-furan-2-carboxylic acid for RNase H function of HIV-1 reverse transcriptase. *Bioorganic and Medicinal Chemistry*.

[131] Su HP, Yan Y, Prasad GS (2010). Structural basis for the inhibition of RNase H activity of HIV-1 reverse transcriptase by RNase H active site-directed inhibitors. *Journal of Virology*.

[132] Shaw-Reid CA, Munshi V, Graham P (2003). Inhibition of HIV-1 ribonuclease H by a novel diketo acid, 4-[5-(benzoylamino)thien-2-yl]-2,4-dioxobutanoic acid. *Journal of Biological Chemistry*.

[133] Tramontano E, Esposito F, Badas R, Di Santo R, Costi R, La Colla P (2005). 6-[1-(4-Fluorophenyl)methyl-1H-pyrrol-2-yl)]-2,4-dioxo-5-hexenoic acid ethyl ester a novel diketo acid derivative which selectively inhibits the HIV-1 viral replication in cell culture and the ribonuclease H activity in vitro. *Antiviral Research*.

[134] Di Santo R, Costi R, Artico M, Tramontano E, La Colla P, Pani A (2003). HIV-1 integrase inhibitors that block HIV-1 replication in infected cells. Planning synthetic derivatives from natural products. *Pure and Applied Chemistry*.

[135] Costi R, Di Santo R, Artico M (2004). 6-Aryl-2,4-dioxo-5-hexenoic acids, novel integrase inhibitors active against HIV-1 multiplication in cell-based assays. *Bioorganic and Medicinal Chemistry Letters*.

[136] Costi R, Di Santo R, Artico M (2004). 2,6-Bis(3,4,5-trihydroxybenzylydene) derivatives of cyclohexanone: novel potent HIV-1 integrase inhibitors that prevent HIV-1 multiplication in cell-based assays. *Bioorganic and Medicinal Chemistry*.

[137] Klumpp K, Hang JQ, Rajendran S (2003). Two-metal ion mechanism of RNA cleavage by HIV RNase H and mechanism-based design of selective HIV RNase H inhibitors. *Nucleic Acids Research*.

[138] Hang JQ, Rajendran S, Yang Y (2004). Activity of the isolated HIV RNase H domain and specific inhibition by N-hydroxyimides. *Biochemical and Biophysical Research Communications*.

[139] Billamboz M, Bailly F, Barreca ML (2008). Design, synthesis, and biological evaluation of a series of 2-hydroxyisoquinoline-1,3*(2H,4H)*-diones as dual inhibitors of human immunodeficiency virus type 1 integrase and the reverse transcriptase RNase H domain. *Journal of Medicinal Chemistry*.

[140] Billamboz M, Bailly F, Lion C (2011). Magnesium chelating 2-hydroxyisoquinoline-1,3(2H, 4H)-diones, as inhibitors of HIV-1 integrase and/or the HIV-1 reverse transcriptase ribonuclease H domain: discovery of a novel selective inhibitor of the ribonuclease H function. *Journal of Medicinal Chemistry*.

[141] Wendeler M, Lee HF, Bermingham A (2008). Vinylogous ureas as a novel class of inhibitors of reverse transcriptase-associated ribonuclease H activity. *ACS Chemical Biology*.

[142] Chung S, Wendeler M, Rausch JW (2010). Structure-activity analysis of vinylogous urea inhibitors of human immunodeficiency virus-encoded ribonuclease H. *Antimicrobial Agents and Chemotherapy*.

[143] Borkow G, Fletcher RS, Barnard J (1997). Inhibition of the ribonuclease H and DNA polymerase activities of HIV- 1 reverse transcriptase by N-(4-tert-butylbenzoyl)-2-hydroxy-1- naphthaldehyde hydrazone. *Biochemistry*.

[144] Arion D, Sluis-Cremer N, Min KL, Abram ME, Fletcher RS, Parniak MA (2002). Mutational analysis of tyr-501 of HIV-1 reverse transcriptase: effects on ribonuclease H activity and inhibition of this activity by N-acylhydrazones. *Journal of Biological Chemistry*.

[145] Sluis-Cremer N, Arion D, Parniak MA (2002). Destabilization of the HIV-1 reverse transcriptase dimer upon interaction with N-acyl hydrazone inhibitors. *Molecular Pharmacology*.

[146] Himmel DM, Sarafianos SG, Dharmasena S (2006). HIV-1 reverse transcriptase structure with RNase H inhibitor dihydroxy benzoyl naphthyl hydrazone bound at a novel site. *ACS Chemical Biology*.

[147] Felts AK, La Barge K, Bauman JD (2011). Identification of alternative binding sites for inhibitors of HIV-1 ribonuclease H through comparative analysis of virtual enrichment studies. *Journal of Chemical Information and Modeling*.

[148] Tatyana K, Francesca E, Luca Z (2009). Inhibition of HIV-1 ribonuclease H activity by novel frangula-emodine derivatives. *Medicinal Chemistry*.

[149] Esposito F, Kharlamova T, Distinto S (2011). Alizarine derivatives as new dual inhibitors of the HIV-1 reverse transcriptase-associated DNA polymerase and RNase H activities effective also on the RNase H activity of non-nucleoside resistant reverse transcriptases. *FEBS Journal*.

[150] Tramontano E, Kharlamova T, Esposito F (2011). Effect of new quinizarin derivatives on both HCV NS5B RNA polymerase and HIV-1 reverse transcriptase associated ribonuclease H activities. *Journal of Chemotherapy*.

[151] Mohan P, Loya S, Avidan O (1994). Synthesis of naphthalenesulfonic acid small molecules as selective inhibitors of the DNA polymerase and ribonuclease H activities of HIV-1 reverse transcriptase. *Journal of Medicinal Chemistry*.

[152] Skillman AG, Maurer KW, Roe DC (2002). A novel mechanism for inhibition of HIV-1 reverse transcriptase. *Bioorganic Chemistry*.

[153] Wang LZ, Kenyon GL, Johnson KA (2004). Novel mechanism of inhibition of HIV-1 reverse transcriptase by a new non-nucleoside analog, KM-1. *Journal of Biological Chemistry*.

[154] Davis CA, Parniak MA, Hughes SH (2011). The effects of RNase H inhibitors and nevirapine on the susceptibility of HIV-1 to AZT and 3TC. *Virology*.

[155] Srivastava S, Sluis-Cremer N, Tachedjian G (2006). Dimerization of human immunodeficiency virus type 1 reverse transcriptase as an antiviral target. *Current Pharmaceutical Design*.

[156] Camarasa MJ, Velázquez S, San-Félix A, Pérez-Pérez MJ, Gago F (2006). Dimerization inhibitors of HIV-1 reverse transcriptase, protease and integrase: a single mode of inhibition for the three HIV enzymes?. *Antiviral Research*.

[157] Grohmann D, Corradi V, Elbasyouny M (2008). Small molecule inhibitors targeting HIV-1 reverse transcriptase dimerization. *ChemBioChem*.

[158] Warren K, Warrilow D, Meredith L, Harrich D (2009). Reverse transcriptase and cellular factors: regulators of HIV-1 reverse transcriptase. *Viruses*.

